# Novel 2-Aryl-1H-Benzimidazole Derivatives and Their Aza-Analogues as Promising Anti-Poxvirus Agents

**DOI:** 10.3390/v18010071

**Published:** 2026-01-04

**Authors:** Valeria Manca, Laura Locci, Roberta Ibba, Laura Sanna, Ilenia Lupinu, Sandra Piras, Gabriele Murineddu, Gabriele Serreli, Roberta Loddo, Rebecca Piras, Luca Virdis, Michela Isola, Vanessa Palmas, Giuseppina Sanna, Antonio Carta

**Affiliations:** 1 Department of Biomedical Science, Section of Microbiology and Virology, University of Cagliari, 09042 Monserrato, Italy; mancavaleria26@gmail.com (V.M.);; 2 Department of Medicine, Surgery and Pharmacy, University of Sassari, 07100 Sassari, Italy; 3 Department of Chemical, Physical, Mathematical and Natural Sciences, University of Sassari, 07100 Sassari, Italy; 4 Department of Biomedical Science, Section of General Pathology, University of Cagliari, 09042 Monserrato, Italy; 5 Department of Biomedical Science, Section of Human Anatomy, University of Cagliari, 09042 Monserrato, Italy

**Keywords:** *Orthopoxvirus*, VV, MPXV, benzimidazoles, antiviral activity, TEER, adsorption assay

## Abstract

Introduction: Despite the impressive progress carried out in the field of biomedical sciences in recent decades, the incidence of emerging and neglected lethal viral infections mainly belonging to the *Coronaviridae*, *Filoviridae*, *Arenaviridae*, *Bunyaviridae*, and *Paramyxoviridae* families has considerably impaired human health. The worldwide vaccination campaign at the end of the 1970s determined the eradication of smallpox. However, the growing number of cases of diseases linked to orthopoxvirus diseases, such as the recent epidemic of monkeypox zoonosis in various countries around the world, has increased the need for knowledge of these viral pathogens. To date, there is no specific treatement for Monkeypox virus (MPXV) infection. However, several antiviral drugs used to treat Smallpox and other viral infections could also be beneficial for Monkeypox disease. In this study we report the design and synthesis of new, variously substituted benzimidazole derivatives and the evaluation of their cytotoxicity and antiviral activity against representatives of the *Orthopoxvirus* genus, Vaccinia Virus (VV), closely related to variola virus and MPXV. Methods: A combination of cell-based assays and experimental techniques was used to investigate the cytotoxicity, antiviral activity, and mechanisms of action of the most interesting compound. Results: In our study, new, variously substituted benzimidazoles showed interesting EC_50_ values against vaccinia and MPXV and a cytotoxic profile in the high micromolar range. Conclusions: Our work shows that the new tested benzimidazole derivatives possess appealing activity and selectivity, accompanied by low cytotoxicity. These results set a valid foundation with which to identify potent and selective anti-Poxvirus agents.

## 1. Introduction

Vaccinia Virus is a member of the family *Poxviridae*, subfamily *Chordopoxvirinae*, and genus *Orthopoxvirus*, which also includes Variola and Monkeypox virus. It is a large, enveloped virus with a linear double-stranded DNA genome and possesses the unique ability to replicate entirely within the cytoplasm.

VV is of major scientific and historical importance, as it has long served as the prototypical poxvirus model for laboratory research. Moreover, it was used as the live vaccine that led to the successful eradication of smallpox in the 1980s (WHO). Four decades after the end of immunization with vaccinia virus, declining population immunity, combined with the wide host range of orthopoxviruses [1], has contributed to the resurgence and increased burden of poxvirus infections (Mpox outbreak [2], particularly the *Monkeypox virus* (MPXV)).

Monkeypox (mpox) is caused by the *Monkeypox virus*, currently considered the most significant orthopoxvirus infection affecting humans [1]. The virus is maintained in African wildlife, primarily rodents, which serve as its natural reservoirs, particularly in Central and West African countries where it is currently endemic [3].

Since 2022, the largest mpox outbreak on record has been spreading worldwide, with over 170.000 confirmed cases globally, primarily in Central and West Africa, with 462 deaths reported across 141 countries. This epidemic has shown a higher number of human cases and greater human-to-human transmission than previously documented [4].

The disease shares clinical features with smallpox, presenting with painful vesiculopustular rashes, often accompanied by fever and lymphadenopathy, and it might progress to severe disease or death [5]. There are two distinct clades of MPXV: clade I (with subclades Ia and Ib) and clade II (with subclades IIa and IIb). The 2022–2023 global outbreak was caused by the clade IIb strain, mainly transmitted through sexual contact. As of 31 July 2025, mpox clade II cases continue to be reported in West Africa, with notable increases around the end of May in Sierra Leone, while most regions outside Africa have shown a sustained decline in transmission.

There are growing outbreaks of clades Ia and Ib affecting the Democratic Republic of Congo and other countries in Africa. Notably, clade Ib was detected outside Africa in August 2024, with multiple cases subsequently reported in Europe by March 2025. Whereas clades I and IIa are primarily zoonoses, clade IIb has exhibited extensive human-to-human spread. Clade Ia and Ib transmission differs from clade IIb, with spread through close contact, including skin rash contact, affecting sexual partners, household members, and healthcare workers [6,7]. Clade Ib is considered to be more virulent and transmissible compared to other strains, which raises concerns about its potential global spread.

The WHO declared a Public Health Emergency of International Concern (PHEIC) on 14 August 2024, which was closed in September 2025. However, the mpox epidemic has continued to spread in the Democratic Republic of the Congo (DRC), Uganda, and neighbouring countries, and the number of new weekly cases is still increasing in the WHO African Region. Consequently, the Africa CDC has designated the MPox pandemic a Public Health Emergency of Continental Security (PHECS).

Two vaccines are currently available for the prophylactic treatment of MPXV infection: modified vaccinia Ankara (MVA; JYNNEOS in the United States and IMVANEX in Europe) and ACAM2000 [8].

In Canada, ACAM2000, along with IMVAMUNE (MVA-BN), is available under an Expanded Access Investigational New Drug (EA-IND) protocol. However, supplies are limited and are prioritized for high-risk population, including immunocompromised individuals [9].

The therapy for MPXV infection consists of life support. Although there is no approved treatment available for mpox infections, tecovirimat (also known as Tpoxx), a potent antiviral drug approved by the FDA for the treatment of smallpox, was used successfully during the 2022 outbreak [10].

Tecovirimat is still in the experimental stage and is being tested for its safety and efficacy in treating mpox. Preliminary findings from the PALM007 trial (initiated in October 2022 in the Democratic Republic of Congo) reported in August 2024 demonstrated no significant reduction in mpox lesion duration with tecovirimat treatment for moderate-to-severe clade I infections [11].

Combination therapies involving tecovirimat and other antivirals or antibiotics may be beneficial for treating specific clinical manifestations, such as systemic, ocular, and oral infections. However, Tecovirimat has a low resistance barrier, and multiple tecovirimat-resistant MPXV strains have been reported [12]. Alternative agents such as brincidofovir (BCV) have been used, although their efficacy remains controversial and their anti-MPXV activity is yet to be clearly defined [13].

Developing effective anti-MPXV agents active against wild-type and drug-resistant variants is urgently needed.

NV-387, a broad-spectrum antiviral, was found to possess strong antiviral activity against an orthopoxvirus in an animal model that is considered an important model to establish potential effectiveness against MPox and Smallpox viruses, as all of these viruses belong to the same family of orthopoxviruses. It is based on a unique host-mimetic nanomedicine platform that targets heparan sulfate proteoglycans (HSPG), a common receptor site used by over 90% of human pathogenic viruses.

NV-387 was found to be safe in Phase I human trials, with no reported adverse events [14].

Antiviral treatment remains a key countermeasure for treating active infections, as well as a prophylactic strategy, particularly for individuals with immunodeficiencies or at high risk of sequelae.

Literature has reported a large use of benzimidazoles in medicine with antimicrobial and antiviral activities [15]. In particular, our group has previously described the broad-spectrum antiviral activity of benzimidazole derivatives [16,17], some of which have also shown interesting EC_50_ values against Vaccinia virus.

In this study, we investigated the potential anti-Vaccinia virus (VV) activity of a selected group of aryl-benzimidazole derivatives. The compounds were synthesized according to previously reported procedures [18,19]; full experimental details are provided in the Supporting Information (Material and Methods—Synthesis, File S1). Compound 3c was found to have compelling anti-vaccinia virus activity, with a cytotoxic profile in the high micromolar range. Cell-based assays provided insight into its potential mode of action. The most interesting derivatives were also tested against a clinical isolate of the MPXV, demonstrating selectivity against the *Poxviridae*. Based on biological data and studies of the mode of action, we hypothesize that the early phase of the viral cycle is a potential target for these compounds. Targeting viral entry minimises the emergence of antiviral resistance and is therefore a superior strategic approach. The structural and functional constraints imposed on the entry machinery, such as capsid stability, fusion protein conformational dynamics, and receptor binding specificity, create significant genetic barriers to resistance.

## 2. Materials and Methods

### 2.1. Cells and Viruses

Cell lines were purchased from the American Type Culture Collection (ATCC) (Manassas, VA, USA). Cell lines supporting the multiplication of orthopoxviruses Vaccinia and Monkeypox were Monkey kidney (Vero-76) [ATCC CRL 1587 *Cercopithecus aethiops*] and the clone Vero E6 [ATCC CRL 1586 *Cercopithecus aethiops*]. Cell cultures were checked periodically for the absence of mycoplasma contamination with MycoTect Kit (Gibco, Thermo Fisher Scientific—Paisley, UK).

Viruses were purchased from the American Type Culture Collection (ATCC) (Manassas, VA, USA), except for the clinical isolate Monkeypox (kindly provided by Professor G. Giammanco, and Prof. Simona De Grazia, University of Palermo). *Poxviridae*: Vaccinia Virus (VV) [vaccine strain Elstree-Lister (ATCC VR-1549)], *Herpesviridae*: Herpesvirus-1 (HSV-1) strain KOS (ATCC VR-1493)]. Viruses were maintained in our laboratory and propagated in appropriate cell lines. The viruses were stored in small aliquots at −80° C until use. All in vitro assays involving the infectious Monkeypox virus were performed in biosafety level-3 (BSL-3) facilities at the Department of Biomedical Sciences of the Cagliari University (Sardinia, Italy).

### 2.2. Cytotoxicity Assays

Vero-76 and Vero E6 cells were seeded in 96-well plates at an initial density of 4 × 10^5^ cells/mL, for Vero-76, Dulbecco’s Modified Eagle Medium (D-MEM) (Thermo Fisher Scientific—UK), while for Vero E6 Eagle’s Minimum Essential Medium (EMEM) (Thermo Fisher Scientific—UK), both with L-glutamine and 25 mg/L kanamycin, supplemented with 10% FBS. Cell cultures were then incubated at 37 °C in a humidified, 5% CO_2_ atmosphere, in the absence or presence of serial dilutions of test compounds. The test medium used for the cytotoxic assay, as well as for the antiviral assay, contained 1% of the appropriate serum. Cell viability was determined at 37 °C by the 3-(4,5-dimethylthiazol-1-yl)-2,5-diphenyltetrazolium bromide (MTT) (Merck KGaA, Darmstadt, Germania) method after 72 h Vero-76 or 120 h for Vero E6 [20]. The cytotoxicity of test compounds was evaluated in parallel with their antiviral activity through the viability of mock-infected, treated cells, as monitored by the MTT method.

### 2.3. Antiviral Assays

Compound’s activity against VV, Monkeypox, and Herpesvirus-1 was determined by plaque reduction assays in infected cell monolayers as described previously [21]. Briefly, a monolayer of Vero-76 cells was grown overnight on a 24-well plate.

The cells were then infected with the proper virus dilutions for 2 h to give 50–100 PFU/well. After removing the unadsorbed virus, 500 µL of medium (D-MEM containing L-glutamine and 4500 mg/L D-glucose, supplemented with 1% inactivated FBS) containing 0.75% methylcellulose and serial dilutions of the test products was added.

Cultures were incubated at 37 °C for 3 (VV, HSV-1) or 6 (Monkeypox) days and then fixed with PBS containing 50% ethanol and 0.8% crystal violet, then washed, and air-dried. The number of plaques in the control (no inhibitor) and experimental wells was then counted. Tecovirimat and Mycophenolic acid were employed as reference compounds in antiviral assays.

### 2.4. Linear Regression Analysis

The extent of cell growth/viability and viral multiplication, at each drug concentration tested, was expressed as a percentage of untreated controls. Concentrations resulting in 50% inhibition (CC_50_ or EC_50_) were determined by linear regression analysis.

### 2.5. TEER (Transepithelial Electrical Resistance) Assay

The cytotoxicity of **3c** was tested on intestinal epithelial cells by estimating the TEER (Transepithelial Electrical Resistance) values as a measure of cell monolayer integrity. Caco-2 cells (ECACC Salisbury, Wiltshire, UK) were cultured in Dulbecco’s modified Eagle’s medium (DMEM), supplemented with 10% heat-inactivated bovine serum, 100 U/mL penicillin, 2 mM l-glutamine, 1% non-essential amino acids, and 100 mg/mL streptomycin at 37 ◦C in a humidified atmosphere of 5% CO_2_, replacing the medium twice a week [22]. All cell culture materials were purchased from Euroclone (Milan, Italy). Caco-2 cells (5 × 10^4^ cells/well), at passage 51–60, were grown in 12 mm i.d. Transwell inserts (polycarbonate membrane, 0.4 μm pore size) (Corning Costar Corp., New York, NY, USA) and culture medium was dispensed both in the apical (0.5 mL) and in the basolateral (1.5 mL) compartment of each well. Resistance was assessed using Millicell–ERS voltohmmeter (Millicell-ERS system, Millipore, Bedford, MA, USA). After cell differentiation (>14 days), only cell monolayers in inserts with TEER values > 300 Ω/cm^2^ were considered for the experiment [22]. Then, **3c** (50 μM) was added to the culture medium, and TEER values were measured at intervals of 3, 18, 24, 36,48, 60, 72 and 96 h and reported as a percentage of the corresponding TEER value at time zero (T = 0). HTX was used as a reference phenolic compound because it is a well-established dietary polyphenol from olive products and is commonly employed as a positive control in studies assessing intestinal barrier function. Human pharmacokinetic studies have demonstrated that HT is absorbed after oral intake, with peak plasma concentrations of free and conjugated forms occurring approximately 30–60 min post-ingestion. This supports the relevance of comparing our compound of interest with a phenolic molecule known to reach physiologically meaningful levels in vivo.

### 2.6. Yield Reduction Assay

Vero 76 cells were inoculated with VV at an m.o.i. of 0.1 in DMEM and tested **3c** at non-cytotoxic concentrations. Following a 2 h adsorption period at 37 °C and 5% CO_2_, the inoculum was removed and replaced with fresh medium containing the same concentration of **3c**. After 72 (VV) hours at 37 °C and 5% CO_2,_ each sample was harvested and diluted with serial passages, starting from 10^−1^ up to 10^−12^. The titer of the serial dilutions of the virus-containing supernatant was determined by standard plaque assay, counting the number of obtained plaques in at least two different dilutions for each concentration.

### 2.7. Virucidal Activity Assay

**3c** (20 μM) was incubated with 1 × 10^5^ PFU/mL of VV at either 4 °C and 37 °C for 1 h. The mixture without the test sample was used as the control. At the end of the incubation period, high dilutions were performed, at which the compound was not active. Virus titers were determined by plaque assay in Vero-76 cells.

### 2.8. Cell Pretreatment Assay

Vero-76 cell monolayers in 24-well plates were incubated with 4 concentrations of the **3c** (100, 20, 4, 0,8 μM) for 2 h. After removal of the compounds and two gentle washes, cells were infected with VV. After the virus had adsorbed to the cells, the inoculum was removed, and the cells were overlaid with 500 µL of medium containing 0.75% methylcellulose. The medium was a mixture of D-MEM with L-glutamine and 4500 mg/L D-glucose, supplemented with 1% inactivated FBS. The cells were then incubated for three days at 37 °C. After this time, the monolayers were fixed with a solution of 50% ethanol and 50% crystal violet. They were then washed and air-dried. The number of plaques in the wells was then counted.

### 2.9. Time Course Assay (ToA)

The confluent monolayers of Vero-76 cells in 24-well tissue culture plates were infected for 1 h at room temperature with VV dilutions to give a final m.o.i. of 1. After adsorption, the monolayers were washed twice with D-MEM medium with L-glutamine, supplemented with 1% inactivated FBS, 1 mM sodium pyruvate, and 0.025 g/L kanamycin (Maintenance Medium) and incubated with the same medium at 5% CO_2_ and 37 °C (time zero). Vero-76 cells were treated with compound **3c** (20 μM, approximately 10 times higher than the EC_50_) or reference (Mycophenolic acid, 20 μM,) for 1 h during the infection period (time 0 or during infection) and at a specific time point, 0 to 2, 2 to 4, 6 to 8. After each incubation period, the monolayers were washed two times with maintenance medium and incubated with fresh medium until 12 h post-infection. Monolayers obtained from the ToA assay were frozen at −80 °C to stop the infection from spreading. Following freeze-thawing, the samples were collected and centrifuged, after which the viral titers were determined by plaque assay. The data were then log-transformed, and the results were presented as means ± standard deviation (SD).

### 2.10. Adsorption Assay

Vero-76 cells grown in a 24-well plate were infected with VV, with an m.o.i. of 0.1, in the presence or absence of compound **3c**. Multiwell plates were incubated for 60 min at 4 °C. Medium containing unabsorbed virus was then removed, cells were washed twice with PBS, and overlayed with medium. Plaques were counted after 72 h of incubation at 37 °C.

### 2.11. Penetration Assay

This penetration assay was performed using the following method [16], with a few modifications: Vero-76 cells were infected with VV at an m.o.i. of 0.1 at 4 °C for 1 h. Cells were washed with ice-cold PBS and then shifted to 37 °C in the presence or absence of compounds. The effect of compounds at different concentrations was tested. After 2 h of virus penetration, the infected cells were then treated with alkaline phosphate-buffered saline (PBS; pH 11) for 1 min to inactivate any viruses that had not penetrated the cells, and then acidic PBS (pH 3) was immediately added to neutralize the mix. The neutralized medium was removed, cells were overlayed with 0.75% methylcellulose in media, and then incubated at 37 °C. The amount of penetrated virus, which survived the acid glycine treatment, was determined by plaque assay after 24 h.

### 2.12. Statistical Analysis

Cell-based experiments were independently repeated at least three times. The data are reported as mean ± standard deviation (SD). The statistical significance was calculated with the Unpaired *t*-test performed in GraphPad Prism, version 10.6.1 (San Diego, CA, USA), * *p* < 0.05, ** *p* < 0.01, *** *p* < 0.001, **** *p* < 0.0001

## 3. Results

### 3.1. Synthesis

Based on previously reported benzimidazole compounds with antipoxvirus activity, a series of derivatives was designed by introducing different small substituents at the 2-position of benzimidazoles and their aza-analogues, including both electron-withdrawing and electron-donating groups on phenyl ring.

**Scheme 1:**
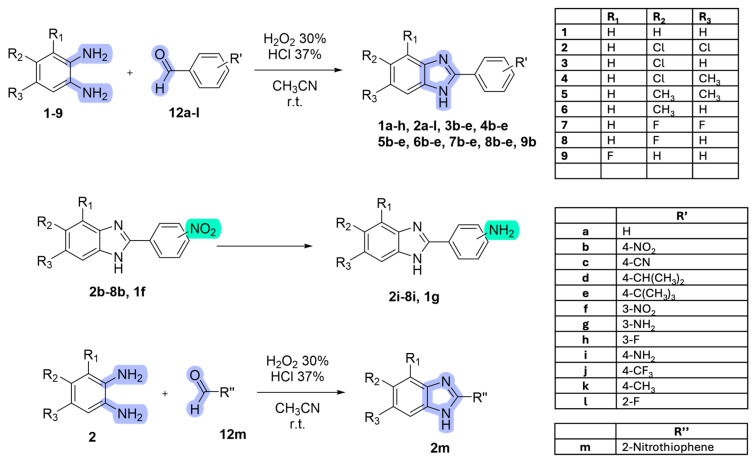
Synthetic route for benzimidazoles. Benzimidazole ring closure was obtained with H_2_O_2_ 30% (7 equation) and HCl 37% (3.5 equation) in MeCN at room temperature; (i): when R_1_ or both R_1_ and R_2_ = Cl, reaction conditions: CH_3_NHNH_2_/EtOH in an autoclave at 100 °C for 48 h; (ii): all the other derivative reaction conditions: NH_2_NH_2_ and Pd/C in EtOH at 80 °C for 1 h.

Derivatives **1a-h**, **2a-l**, **3b-e**, **4b-e**, **5b-e**, **6b-e**, **7b-e**, **8b-e** and **9b** were obtained as described in Scheme 1. Intermediate o-phenylenediamines **1**–**9** (commercially purchased) were mixed with aldehyde **12a-l,** in a ratio of 1:1 and in proper conditions, to obtain derivatives **1a-h**, **2a-l**, **3b-e**, **4b-e**, **5b-e**, **6b-e**, **7b-e**, **8b-e** and **9b**. Derivatives **2b-8b** and **1f** were appropriately reduced to aniline derivatives **2i-8i**, **1g.** Derivative **2m** was obtained starting from intermediate o-phenylenediamine **2** (commercially purchased), which was mixed with aldehyde **12m** in a ratio of 1:1 and proper reagents to obtain derivative **2m**.

**Scheme 2:**
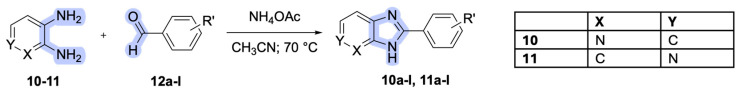
Synthetic route for imidazopyridines. The imidazopyridine ring closure was obtained with ammonium acetate in acetonitrile at 70 °C.

Derivative **10a-l**, **11a-l** were obtained as described in Scheme 2. Intermediate o-pyridinamines **10-11** (commercially purchased) were mixed with aldehyde **12 a-l** (in a ratio of 1:1.1) and ammonium acetate in acetonitrile to obtain derivative **10a-l**, **11a-l**.

### 3.2. Antiviral Assays

The anti-vaccinia virus activity of all newly synthesized derivatives was evaluated in cell-based assays. Several compounds exhibited interesting inhibitory activity, with EC_50_ values in the low micromolar range (i.e., <10 µM; see Table 1). Moderate to low cytotoxicity was also detected for most of the compounds, with CC_50_ values mostly in the high micromolar range (>100 µM) in Vero-76 cells.

Among the assayed compounds, derivative **2b** emerged as non-cytotoxic and displayed a promising anti-VV activity (EC_50_ = 0.06 µM, SI = 600). In contrast, derivatives **2c**, **2e**, and **2j** showed interesting antiviral profiles but were associated with marked cytotoxicity, with CC_50_ values ranging from 6 to 40 µM. Compounds **3c**, **3d**, and **3e** proved to be non-cytotoxic (CC_50_ > 100 µM), exhibiting EC_50_ values of 2.8 (SI = > 36) and 5 (SI = > 20) µM, respectively.

Similarly, derivatives **4b**, **4d**, and **4e** showed comparable antiviral activity (EC_50_ values between 2 and 3 µM); however, only **4b** showed no cytotoxic effects (CC_50_ > 100 µM). Compounds **5b** and **5e** also demonstrated noteworthy antiviral potency, with EC_50_ values of 1 (SI = > 100) and 4 µM (SI = > 25), respectively, and no detectable cytotoxicity (CC_50_ > 100 µM), while **6b** and **6c** were non-cytotoxic, although **6c** exhibited a tenfold lower activity compared to **6b** (EC_50_ = 3 µM).

Derivatives **7b**, **7d**, and **7e** were found to be non-cytotoxic, with EC_50_ values of 9 and 2 µM, respectively. Compound **8d** also displayed antiviral activity without cytotoxic effects (CC_50_ > 100 µM and EC_50_ = 4 µM (SI = >25).

However, as summarized in Table 2 and Table 3, none of the remaining derivatives showed significant anti-VV activity, with EC_50_ values exceeding 10 µM.

From a structure–activity relationship (SARs) perspective, nitro (**b**), isopropyl (**d**), and tert-butyl (**e**) substituents were associated with the highest antiviral activity among the series. Despite their different electronic properties, these moieties consistently modulate the steric and physicochemical environment at the 2-position of the benzimidazole scaffold. In particular, increased steric bulk and/or pronounced electronic effects at this position appear to be beneficial for antiviral potency, suggesting that substitution patterns able to significantly perturb the local environment of the core structure are favored.

Compound **3c** showed the most promising profile and was selected as one of the three best benzimidazole derivatives (**3c**, **4b**, and **7e**) due to its high selectivity index (>36) and the greatest data consistency and reproducibility of the three compounds, with the lowest variability in EC_50_ values.

Hence, derivative **3c** was chosen to be further investigated in order to determine its mode of action. To evaluate the selectivity of the lead compound against poxvirus, the cytotoxicity and antiviral activity of **3c** were tested against an alternative representative of double-stranded DNA viruses: herpes simplex virus 1 (HSV-1).

Notably, **3c** was not able to reduce the number of plaques induced by HSV-1 (Table S1, Supplementary File, File S1), which confirms the active selectivity against the vaccinia virus tested in this study.

### 3.3. TEER Experiment

To ascertain whether derivative **3c** had any toxic effects on human cells, we tested it on differentiated intestinal Caco-2 monolayers, which are commonly used to simulate gut epithelium and evaluate changes in intestinal permeability.

**Figure 1:**
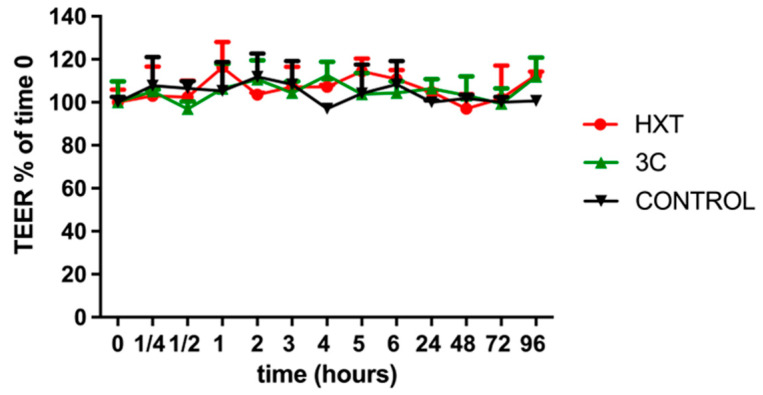
Evaluation of cell monolayer permeabilization as a transepithelial electrical resistance (TEER) assay. Caco-2 cell monolayers were incubated with **3c** (50 µM) and HXT (red circles) as a positive control. In the black cell, control without treatment. Each value represents the mean ± SD of independent experiments (*n* = 3).

The cells were incubated with hydroxytyrosol (HXT) at 60 µM (red) as a positive control, and **3c** derivative at 50 µM (blue). It was observed (Figure 1) that this treatment did not cause any alteration of cell monolayers’ permeability with respect to untreated cells (Control), showing no significant effect on intestinal barrier integrity and no pro-inflammatory effects.

### 3.4. Yield Reduction Assay and Study of the Mechanism of Action

**Figure 2:**
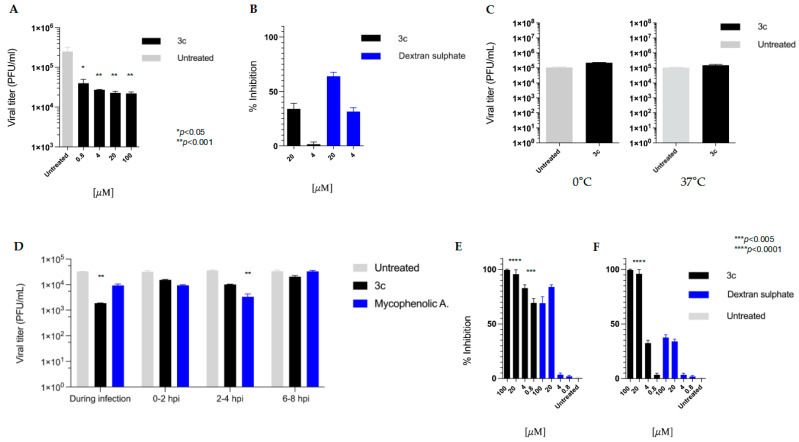
(**A**) Yield of infectious VV produced in infected Vero-76 cells treated with 3c. Vero-76 cells were infected with VV (m.o.i. 0.1). The infected cultures were treated with **3c** at the indicated doses. Viral yields in the culture supernatant were determined by plaque assay at 48 h post-infection. Statistically significant differences are expressed (*p* < 0.05). (**B**) Cell Pretreatment Assay (**C**) and Virucidal effect (expressed as plaque-forming units (PFU/mL) of derivative 3c (15 µM) against VV virions at either 0 °C or 37 °C for 1 h. Dark columns, viral titer for viral and derivative VV solution; Grey columns for the viral titer of the untreated solution. (**D**) Time course assay. Vero-76 cells were inoculated with VV (m.o.i. = 1) and then compound 3c (20 µM) was added at the indicated times. Viral yields were determined by plaque assay. Dark columns, the viral yield for cells treated with 3c derivative; Grey columns, viral yield for control cells; Blue columns, viral yield for cells treated with mycophenolic acid (reference). (**E**) Adsorption Assay. Vero-76 cells were pre-adsorbed for 1 h at 4 °C with viruses at an m.o.i. = 1 in the presence of compound (5 × EC_50_ concentration). Vero-76 cells were inoculated with VV (m.o.i. = 1), and then compound **3c** (100, 20, 4, 0.8 µM) was added at the indicated times. Percentages of inhibition were determined by plaque assay. Grey columns, the percentages of inhibition for control cells; Dark columns, percentage of inhibition for cells treated with **3c** derivative; Blue columns, percentage of inhibition for cells treated with Dextran sulphate (reference). (**F**) Penetration Assay. Dose-dependent of derivative **3c** (Dark columns) on VV penetration. Dextran sulphate (blue columns) was used as an internal control. The results presented were obtained from three independent experiments (two independent experiments for ToA). Data are mean ±SD. T-tests were performed on all groups. **** *p* < 0.0001, *** *p* < 0.001, ** *p* < 0.005, * *p* < 0.05.

To assess the impact of the active compound on the reduction in viral titers, the antiviral activity of compound **3c** was investigated in a yield reduction assay. The concentrations of 100, 20, 4, and 0.8 µM were employed, and a dose-dependent reduction in viral titer was observed (Figure 2A).

### 3.5. Effect of Compound 3c on VV Penetration into Pre-Treated Host Cells

To see if compound **3c** could protect cells from VV infection, a pre-treatment assay was then carried out by incubating Vero-76 cell monolayers (2 h) with different concentrations of **3c** (20μM), 4 μM). We employed dextran sulphate (DS), a broad-spectrum RNA/DNA-enveloped virus attachment inhibitor, as an internal control. The residual unbound compounds were washed off before the Vero-76 cells were infected with VV. We determined the reduction in plaque number after three days.

As shown in Figure 2B, the compound **3c** exhibited modest antiviral activity, achieving less than 40% inhibition even at the highest concentration tested (20 μM, *p* = 0.26).

### 3.6. Virucidal Activity Assay

A virucidal assay was performed to assess the effect of compound **3c** on viral infectivity. No significant differences in the titer of VV treated at two different temperatures were observed (see Figure 2C).

### 3.7. Mode of Action Studies (Time Course, Adsorption and Penetration Assays)

Time of Addition is a method commonly used in virology laboratories to identify the target of a newly discovered antiviral drug in cell culture. It involves comparing the time it takes for the drug to act with that of well-characterised inhibitors. In this assay, we employed mycophenolic acid as a reference compound. As shown in Figure 2D, **3c** exhibited its utmost antiviral activity at the beginning of the infection process (at time 0). As expected, mycophenolic acid, an inhibitor of IMP dehydrogenase, a key enzyme in purine biosynthesis, halted the VV replication cycle during the post-entry phase.

We also characterised the kinetics of the interaction between VV and host cells in the presence of **3c.** We investigated whether **3c** could interfere with the virus binding to target cells by incubating the virus and cell monolayers at 4 °C in the presence of the compound.

Conducting viral adsorption assays at 4 °C is a standard methodology for investigating the initial binding phase between viruses and host cells. This approach exploits the low temperature to permit viral attachment (adsorption) while simultaneously preventing the energy-dependent internalization process (entry), which normally occurs at 37 °C. By maintaining cells at 4 °C during the adsorption phase, we can selectively evaluate potential entry inhibitors. Following the low-temperature incubation, unbound viruses are removed through washing, and the infection process can then proceed at physiological temperatures to assess subsequent stages of viral infection. As shown in Figure 2E, our derivative and dextran sulphate (used as an internal control) were both effective in reducing VV viral binding under these conditions.

We then performed a penetration assay (Figure 2F), scanning the mechanism by which VV entry into cell monolayers is inhibited. We found that compound **3c** could efficiently inhibit viral penetration but only at higher concentrations (20 and 100 µM).

### 3.8. Antiviral Activity Against MPXV

The hit compound **3c** and structurally related compounds **1c, 2c**, **6c**, **11c**, all characterized by a 4′-CN group in the phenyl moiety, were evaluated for anti-MPXV efficacy using the plaque reduction assay. Compound **10c** was not further tested due to the lack of activity against VV. Tecovirimat was employed as the reference MPXV inhibitor. Cell viability was evaluated in parallel at different concentrations to assess cytotoxic effects after 6 days of treatment. Table 4 and Table 5 show that derivative **3c** exhibited the most promising profile with an EC_50_ of 2.7 μM and no cytotoxicity (also when tested for long-term, until 6 days) against both MPXV and VV, demonstrating equipotent and selective antipoxvirus activity.

## 4. Discussion

Due to their range of biological activities, including antiviral, antibacterial, antiparasitic, and anticancer properties, benzimidazole derivatives are key structures in medicinal chemistry. Their antiviral potential was first recognized in the 1960s–1970s with compounds like 2-(α-hydroxybenzyl) benzimidazole (HBB) showing activity against picornaviruses [23]. Literature reports benzimidazole derivatives as active against RNA viruses [24,25] (including influenza, respiratory syncytial virus, enteroviruses, hepatitis C virus, and HIV) as well as DNA viruses (herpesviruses, human cytomegalovirus, and human papillomavirus) [26,27]. This broad antiviral spectrum reflects the versatility of the benzimidazole scaffold in interfering with viral processes through diverse molecular mechanisms.

In our research, we have described the promising anti-poxvirus activity of newly synthesized benzimidazoles. Derivative **3c**, in particular, exhibits a considerably broad-spectrum anti-poxvirus activity against VV and MPXV, with a cytotoxic profile in the high micromolar range. (Table 1, Table 2, Table 3, Table 4 and Table 5).

Compound **3c** was evaluated for epithelial compatibility using Caco-2 cell monolayers to assess permeability and exclude cytotoxicity. Caco-2 cells, derived from human colonic epithelium with in vitro enterocyte differentiation capability, represent an established model for testing nutrients, environmental contaminants, and therapeutic agents.

Our data showed that it did not determine any toxic effects on epithelial cells, resulting safe at the applied concentration (50 μM).

To assess the efficacy of compound **3c** in suppressing viral replication, a yield reduction assay was performed, and a titer reduction (approximately one logarithm) was detected for each tested concentration (100, 20, 4, 0.8 µM).

When the cells were exposed to our compound before being infected, a modest reduction in plaque formation was obtained (less than 40%). However, this test reflects only a single early round of infection, and in this case, a modest inhibitory effect can be exponentially amplified over the multiple rounds, allowing the infection to progress for an extended period, permitting the virus to complete multiple cycles.

Conversely, it is important to exercise caution before attributing that result to the genuine interaction of the compound with the target. In high-throughput screening contexts, similar phenomena are referred to as assay interference [28], where compound effects are observed not because of interaction with the target biology but due to residual chemical activity, aggregation, or non-specific binding that survives standard wash. Even rigorous washing does not guarantee complete removal of all compounds, and these trace amounts may remain on the cell surface and can still modulate events such as viral entry, replication, or plaque formation. Taking these factors into account, the observed ~40% reduction needs further investigations, such as Kinetic washout assays, or Chemical Neutralisation Tests [29].

Although compound **3c** does not directly target the virus, evidence from the time-of-addition assays suggests that it exerts its effect during the initial phases of the infection cycle.

We also observed that **3c** reduced viral adsorption into Vero-76 cells, and it had its utmost effect when added during the infection period. Indeed, as reported in Figure 2F, when tested at higher concentrations, this derivative reduces viral penetration into host cells. The use of benzimidazoles as modulators of the early stages of the replication cycle of various viruses is well established in the literature. Several benzimidazole derivatives [30] have been reported to interfere with cellular or viral processes involved in virus attachment, entry, or the earliest post-entry events, through interactions with viral components [31] or with cellular factors [32] required for replication.

Our experimental data demonstrate that derivative **3c** interferes with early events in the vaccinia replication cycle; however, the precise molecular target remains undefined. We cannot conclusively determine whether the compound acts specifically during viral adsorption or at another early-stage process.

Furthermore, the observed in vitro efficacy has not yet been corroborated through animal model studies. Future work will prioritize identifying the exact viral or cellular target, validating therapeutic efficacy in vivo, and expanding the evaluation to include additional poxviruses to determine whether **3c** possesses broad-spectrum antiviral properties. Interestingly, compounds structurally related to **3c** that were found to be active against VV also displayed selective activity against MPXV, although compound **3c** remained the most potent overall. Our results, therefore, align with established evidence and contribute to expanding the understanding of how benzimidazole-based structures can modulate early events in the viral replication cycle. Additional future research will involve evaluating the combination of **3c** with existing poxvirus therapeutics, such as tecovirimat. These compounds have complementary mechanisms of action, which may result in synergistic activity and offer a strategy to overcome recently documented tecovirimat resistance. These combination studies will be conducted alongside in vivo efficacy assessments in suitable animal models.

## 5. Conclusions

Our research group provided valuable insights into benzimidazole-based antiviral drug design and established this scaffold as a privileged structure for developing new antiviral therapeutics. Benzimidazole derivatives are a versatile and promising class of antiviral agents, with demonstrated activity against a variety of viral families through different mechanisms of action. Our research team’s main challenge at present is to improve our understanding of how these derivatives work and to further enhance the potency, selectivity, and drug-like properties of our benzimidazole derivatives.

This work establishes benzimidazole derivatives as a promising chemical class for the development of next-generation antivirals targeting orthopoxviruses. Among the synthesized compounds, derivative **3c** has demonstrated compelling activity against Vaccinia and Monkeypox viruses, combined with low cytotoxicity and favorable epithelial compatibility. These findings not only validate benzimidazole scaffolds as privileged structures in antiviral drug design but also provide a strategic starting point for translational research aimed at addressing the urgent need for effective treatments against emerging poxvirus infections.

## Figures and Tables

**Table 1:** Cytotoxicity and antiviral activity of selected benzimidazoles against Vaccinia virus expressed as CC_50_ and EC_50_.

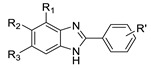						
Number	R_1_	R_2_	R_3_	R′	^a^ Vero-76	^b^ VV
					CC_50_	EC_50_
**1a**	H	H	H	H	>100	>100
**1b**	H	H	H	4-NO_2_	>100	14.4 ± 3.7 (>7)
**1c**	H	H	H	4-CN	>100	22 (>5)
**1d**	H	H	H	4-CH(CH_3_)	55	11 ± 2.1 (5)
**1e**	H	H	H	4-C(CH_3_)_3_	33	12.2 ± 1.8 (3)
**1f**	H	H	H	3-NO_2_	70 ± 3	>70
**1g**	H	H	H	3-NH_2_	>100	>100
**1h**	H	H	H	3-F	>100	>100
**2a**	H	Cl	Cl	H	>100	53 ± 5 (>2)
**2b**	H	Cl	Cl	4-NO_2_	40 (6 dpt)/>100	0.06 ± 0.02 (>1667)
**2c**	H	Cl	Cl	4-CN	11 (6 dpt)/40	0.9 ± 0.1 (45)
**2d**	H	Cl	Cl	4-CH(CH_3_)	20	4.5 ± 0.7 (4.5)
**2e**	H	Cl	Cl	4-C(CH_3_)_3_	9.2	0.9 ± 0.1 (10)
**2i**	H	Cl	Cl	4-NH_2_	16	15.4 ± 2.3 (1)
**2j**	H	Cl	Cl	4-CF_3_	6,3	0.3 ± 0.1 (21)
**2k**	H	Cl	Cl	4-CH_3_	21 (6 dpt)/48	7.8 ± 0.8 (6)
**3b**	H	Cl	H	4-NO_2_	50	3.2 ± 0.8 (16)
**3c**	H	Cl	H	4-CN	>100	2.8 ± 0.07 (>36)
**3d**	H	Cl	H	4-CH(CH_3_)	>100	5.4 ± 0.4 (>18.5)
**3e**	H	Cl	H	4-C(CH_3_)_3_	>100	5.3 ± 0.7 (19)
**3i**	H	Cl	H	4-NH_2_	47	>47
**4b**	H	Cl	CH_3_	4-NO_2_	>100	2.4 ± 0.5 (>42)
**4d**	H	Cl	CH_3_	4-CH(CH_3_)	8 (6 dpt)/25 ± 6	2.4 ± 0.5 (10)
**4e**	H	Cl	CH_3_	4-C(CH3)3	9 (6 dpt)/48.5 ± 3.5	2.1 ± 0.3 (23)
**5b**	H	CH_3_	CH_3_	4-NO_2_	90(6 dpi)/>100	1.0 ± 0.2 (>100)
**5d**	H	CH_3_	CH_3_	4-CH(CH_3_)	34 ± 3	2.7 ± 0.5 (13)
**5e**	H	CH_3_	CH_3_	4-C(CH_3_)_3_	>100	4 ± 0.8 (>25)
**5i**	H	CH_3_	CH_3_	4-NH_2_	71	33 ± 5 (2)
**6b**	H	CH_3_	H	4-NO_2_	>100	3.7 ± 0.7 (>27)
**6c**	H	CH_3_	H	4-CN	>100	36 ± 7 (>3)
**6d**	H	CH_3_	H	4-CH(CH_3_)	37 ± 10	>37
**6e**	H	CH_3_	H	4-C(CH_3_)_3_	>100	>100
**6i**	H	CH_3_	H	4-NH_2_	>100	>100
**7b**	H	F	F	4-NO_2_	>100(6 dpi)/>100	9.5 ± 2.1 (>10.5)
**7d**	H	F	F	4-CH(CH_3_)	>100	9.2 ± 2.3 (>11)
**7e**	H	F	F	4-C(CH_3_)_3_	>100	2.3 ± 1.1 (>44)
**7i**	H	F	F	4-NH2	>100	48.5 ± 3.5 (>2)
**8b**	H	F	H	4-NO_2_	>100	14 ± 0.4 (>7)
**8d**	H	F	H	4-CH(CH_3_)	>100	4.2 ± 1.1 (>24)
**8e**	H	F	H	4-C(CH_3_)_3_	>100	100
**8i**	H	F	H	4-NH_2_	>100	>100
**9b**	F	H	H	4-NO_2_	>100	>100
Tecovirimat					>100	0.06 ± 0.03
Mycophenolic Acid (M5255)					80	1.7 ± 0.2

^a^ Compound concentration (μM) required to reduce the viability of mock-infected Vero 76 monolayers by 50%, as determined by the MTT method. ^b^ Compound concentration (μM) required to reduce the plaque number by 50% in Vero-76 monolayers infected by Vaccinia virus. Selectivity Index (SI).

**Table 2:** Cytotoxicity and antiviral activity of selected benzimidazoles against Vaccinia virus expressed as CC_50_ and EC_50_.

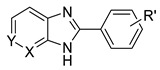					
Number	X	Y	R′	^a^ Vero-76	^b^ VV
				CC_50_	EC_50_
**10c**	N	C	4-CN	>100	>100
**10h**	N	C	3-F	64 ± 5.6	>64
**10j**	N	C	4-CF_3_	10 ± 0.8	>10
**11b**	C	N	4-NO_2_	>100	18 (>6)
**11c**	C	N	4-CN	>100	18 (>6)
**11h**	C	N	3-F	>100	>100
**11L**	C	N	2-F	>100	>100

^a^ Compound concentration (μM) required to reduce the viability of mock-infected Vero 76 monolayers by 50%, as determined by the MTT method. ^b^ Compound concentration (μM) required to reduce the plaque number by 50% in Vero-76 monolayers infected by Vaccinia virus. Selectivity Index (SI).

**Table 3:** Cytotoxicity and antiviral activity of selected benzimidazoles against Vaccinia virus expressed as CC_50_ and EC_50_.

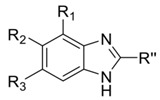						
Number	R_1_	R_2_	R_3_	R′′	^a^ Vero-76	^b^ VV
					CC_50_	EC_50_
**2m**	H	Cl	Cl	2-nitrothiophene	>100 (6 dpi)/>100	30 ± 4 (>3)

^a^ Compound concentration (μM) required to reduce the viability of mock-infected Vero 76 monolayers by 50%, as determined by the MTT method. ^b^ Compound concentration (μM) required to reduce the plaque number by 50% in Vero-76 monolayers infected by Vaccinia virus. Selectivity Index (SI).

**Table 4:** Cytotoxicity and antiviral activity of selected compounds against MPXV expressed as CC_50_ and EC_50_.

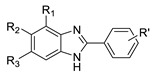						
Number	R_1_	R_2_	R_3_	R′	^a^ Vero-76	^c^ MPXV
					CC_50_	EC_50_
**1c**	H	H	H	4-CN	>100	>100
**2c**	H	Cl	Cl	4-CN	11 (6 dpt)/40	>11
**3c**	H	Cl	H	4-CN	>100	2.7 ± 1 (>37)
**6c**	H	CH_3_	H	4-CN	>100	40 ± 10 (>2.5)
Tecovirimat					>100	0.13 ± 0.05

^a^ Compound concentration (μM) required to reduce the viability of mock-infected Vero 76 monolayers by 50%, as determined (6 dpi) by the MTT method. ^c^ Compound concentration (μM) required to reduce the plaque number by 50% in Vero-76 monolayers infected by Monkeypox virus. Selectivity Index (SI).

**Table 5:** Cytotoxicity and antiviral activity of selected compounds against MPXV expressed as CC_50_ and EC_50_.

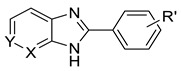					
Number	X	Y	R′	^a^ Vero-76	^c^ MPXV
				CC_50_	EC_50_
**10c**	N	C	4-CN	>100	-
**11c**	C	N	4-CN	>100	8 (>12.5)
Tecovirimat				>100	0.13 ± 0.05

^a^ Compound concentration (μM) required to reduce the viability of mock-infected Vero 76 monolayers by 50%, as determined (6 dpi) by the MTT method. ^c^ Compound concentration (μM) required to reduce the plaque number by 50% in Vero-76 monolayers infected by Monkeypox virus. Selectivity Index (SI).

## Data Availability

The original contributions presented in this study are included in the article/Supplementary Material. Further inquiries can be directed to the corresponding authors.

## Supplementary Materials

The following supporting information can be downloaded at https://www.mdpi.com/article/10.3390/v18010071/s1, Table S1: Cytotoxicity and antiviral activity of 3c against Herpesvirus (HSV-1); File S1: Materials and Methods—Synthesis and characterisation of compounds.
