# Supplementary material for: Novel 2-Aryl-1H-Benzimidazole Derivatives and Their Aza-Analogues as Promising Anti-Poxvirus Agents
Source: Viruses. 2026 Jan 4;18(1):71. doi: 10.3390/v18010071 (PMC12846358; doi:10.3390/v18010071)
Supplement: Supplementary file 1 [file viruses-18-00071-s001.zip › viruses-4020799-supplementary.pdf]

Supporting Information

Novel 2-Aryl-1H-benzimidazole derivatives and their aza-analogues as promising anti-Poxvirus agents

Valeria Manca [1], Laura Locci [1], Roberta Ibba [2], Laura Sanna [3], Ilenia Lupinu [3], Sandra Piras [2], Gabriele Murineddu [2], Gabriele Serreli [4], Roberta Loddo [1], Rebecca Piras [1], Luca Virdis [1], Michela Isola [5], Vanessa Palmas [1], Giuseppina Sanna [1*§] and Antonio Carta [*2§]

Table S1. Cytotoxicity and antiviral activity of 3c against Herpesvirus (HSV-1) expressed as CC_{50} and EC_{50}.

Number	R_1	R_2	R_3	R''	[a]Vero-76	[b]HSV-1
					CC_{50}	EC_{50}
3c	H	Cl	H	4-CN	>100	>100
Acicloguanosine	-	-	-	-	>100	2

[a]Compound concentration (μM) required to reduce the viability of mock-infected Vero 76 monolayers by 50%, as determined by the MTT method.

[b]Compound concentration (μM) required to reduce the plaque number by 50% in Vero-76 monolayers infected by Herpesvirus-1. The results presented were obtained from three independent experiments. Variation among duplicate samples was less than 15%.

File S1:
Material and Methods – Synthesis

1. Synthesis

1. General Synthetic Strategies

All starting materials were purchased by Sigma-Aldrich, Across Organics, and Carlo Erba producers. Benzimidazole ring closure was carried out as described by Bahrami et al. [15] by mixing o-phenylenediamines (1 eq.) and aldehydes (1 eq.) in a ratio of 1:1 in acetonitrile as the solvent, with H_2O_2 30% (7 eq.) and HCl 37% (3.5 eq.) added in turn, reaction is let stir at room temperature till completion of reaction measured through TLC. The product was obtained as precipitated solid that has been filtered off with vacuum, washed with acetonitrile and with water till neutral pH of filtrate. The pure products were obtained via crystallization using ethanol (**Method A**). Imidazopyridines ring closure was carried out as described by Hashem et al. [16] by mixing o-pyridinamines (1 eq.) and aldehydes (1.1 eq.) in ratio 1:1.1 in acetonitrile as the solvent, with ammonium acetate (4 eq.) added at 70°C till completion of reaction measured through TLC. Upon resting at room temperature, the solid product precipitated was filtered off and washed with acetonitrile. The pure products were obtained via crystallization using ethanol (**Method B**). Nitrogen groups reductions to obtain amine derivatives were performed in ethanol by three different routes as follows: **Method C**) in autoclave at 100°C with methylhydrazine in case of chlorine-containing derivatives, to avoid displacement of chlorine; **Method D**) at room temperature with H_2 and Pd/C as catalyst; **Method E**) at 80°C with hydrazine and Pd/C as catalyst. Products were purified by crystallization from ethanol or methanol, or by flash chromatography using appropriate elution mixture.

2. Chemical Characterization

Nuclear Magnetic Resonance (NMR) spectra were registered in solutions in deuterated acetone, DMSO or DMSO+TFA and were recorded with a Bruker Avance III 400 NanoBay (400 MHz) instrument. ^{1}H NMR chemical shifts are reported in parts per million (ppm) downfield from tetramethyl silane (TMS) used as internal standard. Chemical shift values are reported in ppm (δ) and coupling constants (J) are re-

ported in Hertz (Hz). Signal multiplicities are represented as s (singlet), ws (wide singlet), d (doublet), dd (doublet of doublets), ddd (doublet of doublet of doublets), t (triplet), td (triplet of doublets), q (quadruplet) and m (multiplet). ^{13}C NMR chemical shifts are reported in downfield from tetramethylsilane (TMS) used as internal standard. Suitable method among APT (Attached Proton Test), jmod (J-modulated spin-echo for X-nuclei coupled to H-1 to determine number of attached protons) and ZGPG30 (1D sequence with power-gated decoupling using 30-degree flip angle) was selected for each compound. Two-dimensional NMR experiments HSQC (Heteronuclear Single Quantum Coherence) and HMBC (Heteronuclear Multiple Bond Correlation) were performed to correctly assign the peaks.

The solutions for high resolution ESI-MS measurements were prepared by dissolving the solid compounds in HPLC acetonitrile to obtain a concentration of 1.0-2.0 ppm. Mass spectra in the positive-ion mode were obtained on a Q Exactive Plus Hybrid Quadrupole-Orbitrap (Thermo Fisher Scientific) mass spectrometer. The solutions were infused at a flow rate of 5.00 µl/min into the ESI chamber. The spectra were recorded in the m/z range 150–800 at a resolution of 140 000 and accumulated for at least 2 min in order to increase the signal-to-noise ratio. The instrumental conditions used for the measurements were as follows: spray voltage 2300 V, capillary temperature 250°C, sheath gas 10 (arbitrary units), auxiliary gas 3 (arbitrary units), sweep gas 0 (arbitrary units), and probe heater temperature 50°C. HR-ESI-MS spectra were analyzed by using Thermo Xcalibur 3.0.63 software (Thermo Fisher Scientific), and the average deconvoluted monoisotopic masses were obtained through the Xtract tool integrated in the software.

The solutions for ESI-MS measurements were prepared at a concentration of 10 ppm by dissolving and serially diluting the solid compounds in HPLC acetonitrile. Mass spectra in the positive-ion mode were obtained on an Applied Biosystem MDS Sciex API 2000 triple quadrupole (Concord, Ontario, Canada), equipped with an electrospray ionization (ESI) interface. The solutions were infused at a flow rate of 5.00 µL/min into the ESI chamber. The spectra were recorded in the m/z range of 150–600. The instrumental conditions used for the measurements were as follows: ion spray voltage at 2300 V, curtain gas (CUR) and collision gas (CAD) at 6 arbitrary units. ESI-MS spectra were analyzed by using an Applied Biosystem Analyst version 1.6 software.

Retention factors (Rf) were measured by Thin Layer Chromatography (TLC) using Merck F-254 commercial plates.

3. Synthesis, Purification and Characterization of Benzimidazole derivatives (Series 1 to 9), Imidazo[4,5-b]pyridine derivatives (Series 10) and Imidazo[4,5-c]pyridine derivatives (Series 11).

1. Synthesis, Purification and Characterization of Unsubstituted Benzimidazole Derivatives (Series 1).

2-(4-nitrophenyl)-1H-benzo[d]imidazole (**1b**), 4-(1H-benzo[d]imidazol-2-yl)benzonitrile (**1c**), 2-(4-isopropylphenyl)-1H-benzo[d]imidazole (**1d**), 2-(4-(tert-butyl)phenyl)-1H-benzo[d]imidazole (**1e**), 2-(3-nitrophenyl)-1H-benzo[d]imidazole (**1f**), 3-(1H-benzo[d]imidazol-2-yl)aniline (**1g**), 2-(3-fluorophenyl)-1H-benzo[d]imidazole (**1h**) were obtained by using proper starting material, through **Method A**.

2. Synthesis, Purification and Characterization of 5,6-dichloro Benzimidazole Derivatives (Series 2).

5,6-dichloro-2-phenyl-1H-benzo[d]imidazole (**2a**), 5,6-dichloro-2-(4-nitrophenyl)-1H-benzo[d]imidazole (**2b**), 4-(5,6-dichloro-1H-benzo[d]imidazol-2-yl)benzonitrile (**2c**), 5,6-dichloro-2-(4-isopropylphenyl)-1H-benzo[d]imidazole (**2d**), 2-(4-(tert-butyl)phenyl)-5,6-dichloro-1H-benzo[d]imidazole (**2e**), 5,6-dichloro-2-(4-(trifluoromethyl)phenyl)-1H-benzo[d]imidazole (**2j**), 5,6-dichloro-2-(p-tolyl)-1H-benzo[d]imidazole (**2k**), 5,6-dichloro-2-(5-nitrothiophen-2-yl)-1H-benzo[d]imidazole (**2m**) were obtained by using proper starting material, through Method A. Amine 4-(5,6-dichloro-1H-benzo[d]imidazol-2-yl)aniline (**2i**) was obtained via reduction in corresponding nitro derivative **2b** through **Method C**.

3. Synthesis, Purification and Characterization of 5-chloro Benzimidazole Derivatives (Series 3).

5-chloro-2-(4-nitrophenyl)-1H-benzo[d]imidazole (**3b**), 4-(5-chloro-1H-benzo[d]imidazol-2-yl)benzonitrile (**3c**), 5-chloro-2-(4-isopropylphenyl)-1H-benzo[d]imidazole (**3d**), 2-(4-(tert-butyl)phenyl)-5-chloro-1H-benzo[d]imidazole (**3e**), were obtained by using proper starting material, through **Method A**.

Amine 4-(5-chloro-1H-benzo[d]imidazol-2-yl)aniline (**3i**) was obtained via reduction of corresponding nitro derivative **3b** through **Method C**.

4. Synthesis, Purification and Characterization of 6-chloro-7-methyl Benzimidazole Derivatives (Series 4).

6-chloro-7-methyl-2-(4-nitrophenyl)-1H-benzo[d]imidazole (**4b**), 2-(4-(tert-butyl)phenyl)-6-chloro-7-methyl-1H-benzo[d]imidazole (**4d**), 6-chloro-2-(4-isopropylphenyl)-7-methyl-1H-benzo[d]imidazole (**4e**) were obtained by using proper starting material, through **Method A**.

5. Synthesis, Purification and Characterization of 5,6-dimethyl Benzimidazole Derivatives (Series 5).

5,6-dimethyl-2-(4-nitrophenyl)-1H-benzo[d]imidazole (**5b**), 2-(4-isopropylphenyl)-5,6-dimethyl-1H-benzo[d]imidazole (**5d**), 2-(4-(tert-butyl)phenyl)-5,6-dimethyl-1H-benzo[d]imidazole (**5e**) were obtained by using proper starting material, through **Method A**. Amine 4-(5,6-dimethyl-1H-benzo[d]imidazol-2-yl)aniline (**5i**) was obtained via reduction of corresponding nitro derivative **5b** through **Method D**.

6. Synthesis, Purification and Characterization of 5-methyl Benzimidazole Derivatives (Series 6).

5-methyl-2-(4-nitrophenyl)-1H-benzo[d]imidazole (**6b**), 4-(5-methyl-1H-benzo[d]imidazol-2-yl)benzonitrile (**6c**), 2-(4-isopropylphenyl)-5-methyl-1H-benzo[d]imidazole (**6d**), 2-(4-(tert-butyl)phenyl)-5-methyl-1H-benzo[d]imidazole (**6e**), were obtained by using proper starting material, through **Method A**. Amine 4-(5-methyl-1H-benzo[d]imidazol-2-yl)aniline (**6i**) was obtained via reduction of corresponding nitro derivative **6b** through **Method D**.

7. Synthesis, Purification and Characterization of 5,6-difluoro Benzimidazole Derivatives (Series 7).

5,6-difluoro-2-(4-nitrophenyl)-1H-benzo[d]imidazole (**7b**), 5,6-difluoro-2-(4-isopropylphenyl)-1H-benzo[d]imidazole (**7d**), 2-(4-(tert-butyl)phenyl)-5,6-difluoro-1H-benzo[d]imidazole (**7e**), were obtained by using proper starting material, through **Method A**. Amine 4-(5,6-difluoro-1H-benzo[d]imidazol-2-yl)aniline (**7i**) was obtained via reduction of corresponding nitro derivative **7b** through **Method E**.

8. Synthesis, Purification and Characterization of 5-fluoro Benzimidazole Derivatives (Series 8).

5-fluoro-2-(4-nitrophenyl)-1H-benzo[d]imidazole (**8b**), 5-fluoro-2-(4-isopropylphenyl)-1H-benzo[d]imidazole (**8d**), 2-(4-(tert-butyl)phenyl)-5-fluoro-1H-benzo[d]imidazole (**8e**), were obtained by using proper starting material, through **Method A**. Amine 4-(5-fluoro-1H-benzo[d]imidazol-2-yl)aniline (**8i**) was obtained via reduction of corresponding nitro derivative **8b** through **Method E**.

9. Synthesis, Purification and Characterization of 4-fluoro Benzimidazole Derivative (Series 9).

4-fluoro-2-(4-nitrophenyl)-1H-benzo[d]imidazole (**9b**) was obtained by using proper starting material, through **Method A**.

10. Synthesis, Purification and Characterization of Imidazo[4,5-b]pyridine Derivatives (Series 10).

4-(3H-imidazo[4,5-b]pyridin-2-yl)benzonitrile (**10c**), 2-(4-(trifluoromethyl)phenyl)-3H-imidazo[4,5-b]pyridine (**10j**), 2-(3-fluorophenyl)-3H-imidazo[4,5-b]pyridine (**10h**) were obtained by using proper starting material, through **Method B**.

11. Synthesis, Purification and Characterization of Imidazo[4,5-c]pyridine Derivatives (Series 11).

4-(3H-imidazo[4,5-c]piridin-2yl)benzonitrile (**11c**), 2-(3-fluorophenyl)-3H-imidazo[4,5-c]pyridine (**11h**), 2-(2-fluorophenyl)-3H-imidazo[4,5-c]pyridine (**11l**) were obtained by using proper starting material, through **Method B**.

2. *Experimental characterization*

2-(4-nitrophenyl)-1H-benzo[d]imidazole (**1b**)

Compound **1b** ($C_{13}H_9N_3O_2$, MW 239.229) was obtained with a total yield of 44%; TLC ($CHCl_3/CH_3OH$ 95/5): R_f 0.72. ^{1}H-NMR (DMSO-*d*$_6$): *δ* 8.49 (4H, s, H-2',3',5',6'), 7.76 (2H, m, H-4,7), 7.41 (2H, m, H-5,6). ^{13}C-NMR (jmod, DMSO- *d*$_6$): δ 148.45 (C), 148.24 (C), 136.98 (2C), 133.63 (C), 128.10 (2CH), 124.41 (2CH), 124.18 (2CH), 115.18 (2CH). HR-ESI-MS (*m/z*): calcd. for $C_{13}H_9N_3O_2$ 240.07675, found 240.07655 $[M+H]^+$.

4-(1H-benzo[d]imidazol-2-yl)benzonitrile (**1c**)

Compound **1c** ($C_{14}H_9N_3$, MW 219.25) was obtained with a total yield of 28%; TLC ($CHCl_3/CH_3OH$ 95/5): R_f 0.6. ^{1}H NMR (DMSO-*d*$_6$ + TFA-*d*): δ 8.37 (2H, d, J=8.4 Hz, H-2', 6'), 8.23-8.16 (2H, m, H-3',5'), 7.91-7.89 (2H, m, H-4, 7), 7.63-7.60 (2H, m, H-5, 6). ^{13}C NMR (DMSO-*d*$_6$ + TFA-*d*): δ 146.95 (C), 133.24 (2CH), 131.57 (2C), 128.57 (2CH), 126.90 (C), 126.44 (2CH), 117.55 (C), 115.37 (C), 114.06 (2CH). ESI-MS (*m/z*): 219.8 $[M+H]^+$.

2-(4-isopropylphenyl)-1H-benzo[d]imidazole (**1d**)

Compound **1d** ($C_{16}H_{16}N_2$, MW 236.312) was obtained with a total yield of 88%; TLC (PS/EA 7/3): R_f 0.62. ^{1}H-NMR (DMSO- *d*$_6$): *δ* 8.11 (2H, d, J= 8.6 Hz, H-2',6'), 7.66 (1H, d, J= 7 Hz, H-4), 7.61 (2H, d, J= 8.6 Hz, H-3',5'), 7.54 (1H, d, J= 7 Hz, H-7), 7.21 (2H, m, H-5,6), 1.34 (9H, s, 3CH3). ^{13}C-NMR (jmod, DMSO- *d*$_6$): *δ* 152.49 (C), 151.18 (C), 142.98 (C), 136.94 (C), 128.42 (C), 127.19 (2CH), 125.79 (2CH), 123.55 (CH), 122.50 (CH), 117.68 (CH), 111.16 (CH), 33.46 (CH), 22.99 ($2CH_3$). HR-ESI-MS (*m/z*): calcd for $C_{16}H_{16}N_2$ 236.13135, found 237.13862 $[M+H]^+$.

2-(4-(tert-butyl)phenyl)-1H-benzo[d]imidazole (**1e**)

Compound **1e** ($C_{17}H_{18}N_2$, MW 250.338) was obtained with a total yield of 65%; TLC (PS/EA 7/3): R_f 0.5. ^{1}H-NMR (DMSO- *d*$_6$): *δ* 8.11 (2H, d, J= 8.4 Hz, H-2',6'), 7.65 (1H, d, J= 7.2 Hz, H-4), 7.57 (2H, d, J= 8.4 Hz, H-3',5'), 7.52 (1H, d, J= 6.8 Hz, H-7), 7.19 (2H, m, H-5,6), 1.34 (9H, s, 3CH3). ^{13}C-NMR (jmod, DMSO- *d*$_6$): *δ* 152.53 (C), 151.24 (C), 143.82 (C), 134.92 (C), 127.41 (C), 126.19 (2CH), 125.70 (2CH), 122.29 (CH), 121.50 (CH), 118.69 (CH), 111.16 (CH), 34.56 (C), 30.96 ($3CH_3$). HR-ESI-MS (*m/z*): calcd. for $C_{17}H_{18}N_2$ 251.15428, found 251.15436 $[M+H]^+$.

2-(3-nitrophenyl)-1H-benzo[d]imidazole (**1f**)

Compound 1f ($C_{13}H_9N_3O_2$, MW 239.233) was obtained with a total yield of 68%; ($CHCl_3/CH_3OH$ 97/3): R_f 0.58. ^{1}H-NMR (400 MHz, DMSO- *d*$_6$) δ 9.08 (d, J = 2.1 Hz, 1H, H-2'), 8.67 (d, J = 7.8 Hz, 1H, H-6'), 8.44 (dd, J = 8.1, 2.3 Hz, 1H, H-4'), 7.95 (t, J = 8.0 Hz, 1H, H-5'), 7.77 (dd, J = 6.1, 3.2 Hz, 2H, H-4,7), 7.43 (dd, J = 6.1, 3.2 Hz, 2H, H-5,6). ^{13}C-NMR (jmod, DMSO- *d*$_6$): δ 148.32 (2C), 147.86 (C), 135.38 (C), 133.32 (2CH), 131.05 (CH), 128.16 (C), 125.88 (CH), 124.67 (CH), 121.94 (2CH), 114.82 (CH). ESI-MS *(m/z):* 240.1 $[M+H]^+$.

3-(1H-benzo[d]imidazol-2-yl)aniline (**1g**)

Compound **1g** ($C_{13}H_{11}N_3$, MW 209.251) was obtained with total yield of 92%; TLC ($CHCl_3/CH_3OH$ 95/5): R_f 0.38. ^{1}H-NMR (400 MHz, DMSO- *d*$_6$) δ 7.56 (dt, *J* = 7.0, 3.5 Hz, 2H, H-7, H-4), 7.43 (t, *J* = 2.0 Hz, 1H, H-5'), 7.28 (dt, *J* = 7.6, 1.3 Hz, 1H, H-6'), 7.24 – 7.14 (m, 3H, H-5, H-6, H-2'), 6.69 (ddd, *J* = 7.9, 2.4, 1.0 Hz, 1H, H-4'). ^{13}C-NMR (jmod, DMSO- *d*$_6$): δ 150.05 (2C), 147.64 (C), 133.47 (C), 130.65 (2CH), 125.65 (CH), 125.29 (C), 119.94 (CH), 117.32 (CH) 114.58 (2CH), 113.83 (CH)**.** ESI-MS *(m/z):* 210.2 $[M+H]^+$.

2-(3-fluorophenyl)-1H-benzo[d]imidazole (**1h**)

Compound **1h** ($C_{13}H_9N_2F$, MW 212.226) was obtained with a total yield of 84%; TLC ($CHCl_3/CH_3OH$ 97/3): R_f 0.66. ^{1}H-NMR (400 MHz, DMSO-*d*$_6$) δ 8.04 (d, $J_{H\text{-}F\ ortho}$ = 7.8 Hz, 1H, H-2'), 7.97 (dt, J = 10.3, 2.0 Hz, 1H, H-4'), 7.69 (d, J = 7.8 Hz,

1H, H-6'), 7.66 – 7.53 (m, 2H, H-4, H-7), 7.35 (td, J_{H-H}= 8.6, J=$_{H-F\ meta}$ 2.6 Hz, 1H, H-5'), 7.29-7.18 (m, 2H, H-5, H-6). ESI-MS *(m/z):* 212.9 $[M+H]^+$.

5,6-dichloro-2-phenyl-1H-benzo[d]imidazole (**2a**)

Compound **2a** ($C_{13}H_8Cl_2N_2$, MW 263.122) was obtained with a total yield of 65%; TLC (PS/EA 7/3): R_f 0.15. ^{1}H-NMR (DMSO- d_6): *δ* 8.19 (2H, d, J= 6.4 Hz, H-2',6'), 7.90 (2H, s, H-4,7), 7.60 (3H, m, H-3',4',5'). ^{13}C-NMR (jmod, DMSO- d_6): *δ* 153.23 (C), 137.36 (C), 131.28 (CH), 129.20 (3CH), 127.79 (C), 127.62 (C), 127.06 (2CH), 125.48 (C), 125.37 (C), 116.13 (CH). HR-ESI-MS (*m*/*z*): calcd. for $C_{13}H_8Cl_2N_2$ 263.01373, found 263.01422 $[M+H]^+$.

5,6-dichloro-2-(4-nitrophenyl)-1H-benzo[d]imidazole (**2b**)

Compound **2b** ($C_{13}H_7Cl_2N_3O_2$, MW 308.120) was obtained with a total yield of 69%; TLC (PS/EA 7/3): R_f 0.58. ^{1}H-NMR (DMSO- d_6): δ 8.40 (4H, ws, H-2',3',5',6'), 7.91 (2H, s, H-4,7). ^{13}C-NMR (jmod, DMSO- d_6): δ 151.50 (C), 148.25 (C), 188.80 (2C), 134.88 (C), 127.42 (2CH), 125.47 (2C), 124.32 (2CH), 116.83 (2CH). HR-ESI-MS (*m*/*z*): calcd. for $C_{13}H_7Cl_2N_3O_2$ 307.99881, found 307.99899 $[M+H]^+$.

4-(5,6-dichloro-1H-benzo[d]imidazol-2-yl)benzonitrile (**2c**)

Compound **2c** ($C_{14}H_7Cl_2N_3$, MW 288.132) was obtained with a total yield of 79%; TLC (PS/EA 7/3): R_f 0.47. ^{1}H-NMR (DMSO- d_6): *δ* 8.30 (2H, d, J= 8.4 Hz, H-2',6'), 8.03 (2H, d, J= 8.4 Hz, H-3',5'), 7.89 (2H, s, H-4,7). ^{13}C-NMR (jmod, DMSO- d_6): *δ* 151.89 (C), 140.63 (C), 133.32 (C), 132.99 (2CH), 131.33 (C), 127.94 (CH), 127.54 (2CH), 126.16 (CH), 125.21 (C), 124.82 (C), 118.42 (C), 112.51 (C). HR-ESI-MS (*m*/*z*): calcd. for $C_{14}H_7Cl_2N_3$ 288.00898, found 288.00891 $[M+H]^+$.

5,6-dichloro-2-(4-isopropylphenyl)-1H-benzo[d]imidazole (**2d**)

Compound **2d** ($C_{16}H_{14}Cl_2N_2$, MW 305.202) was obtained with a total yield of 87%; TLC (PS/EA 7/3): R_f 0.75. ^{1}H-NMR (DMSO- d_6): *δ* 8.19 (d, 2H, J= 8 Hz, H-2',6'), 7.96 (s, 2H, H-4,7), 7.53 (d, 2H, J= 8 Hz, H-3',5'), 3.01 (q, 1H, J= 6.8 Hz, CH), 1.26 (d, 6H, J= 6.8 Hz, $2CH_3$). ^{13}C-NMR (jmod, DMSO- d_6): *δ* 153.19 (C), 152.38 (C), 135.09 (2C), 127.70 (2CH), 127.60 (2CH), 126.43 (2C), 123.42 (C), 115.73 (2CH), 33.47 (CH), 23.46 (2CH3). HR-ESI-MS (*m*/*z*): calcd. for $C_{16}H_{14}Cl_2N_2$ 305.06068, found 305.06085 $[M+H]^+$.

2-(4-(tert-butyl)phenyl)-5,6-dichloro-1H-benzo[d]imidazole (**2e**)

Compound **2e** ($C_{17}H_{16}Cl_2N_2$, MW 319.228) was obtained with a total yield of 63%; TLC (PS/EA 7/3): R_f 0.84. ^{1}H-NMR (DMSO- d_6): *δ* 8.20 (d, 2H, J= 8.4 Hz, H-2',6'), 7.96 (s, 2H, H-4,7), 7.68 (d, 2H, J= 8.4 Hz, H-3',5'), 1.35 (s, 9H, $3CH_3$). ^{13}C-NMR (jmod, DMSO- d_6): *δ* 154.82 (C), 152.88 (C), 136.28 (2C), 127.12 (2CH), 126.15 (2CH), 125.82 (2C), 124.09 (C), 115.89 (2CH), 34.81 (C), 30.80 ($3CH_3$). HR-ESI-MS (*m*/*z*): calcd. for $C_{17}H_{16}Cl_2N_2$ 319.07633, found 319.07657 $[M+H]^+$.

4-(5,6-dichloro-1H-benzo[d]imidazol-2-yl)aniline (**2i**)

Compound **2i** ($C_{13}H_9Cl_2N_3$, MW 278.137) was obtained with a total yield of 97%; TLC ($CHCl_3$/CH_3OH 95/5): R_f 0.28. ^{1}H-NMR (DMSO- d_6): δ 7.83 (2H, d, J= 7.6 Hz, H-2',6'), 7.78 (1H, s, H-4), 7.61 (1H, s, H-7), 6.67 (2H, d, J= 7.6 Hz, H-3',5'), 5.72 (2H, s, NH2). ^{13}C-NMR (jmod, DMSO- d_6): δ 155.21 (C), 151.22 (C), 143.89 (C), 134.56 (C), 128.12 (2CH), 123.46 (C), 123.28 (C), 118.78 (CH), 113.48 (2CH), 116.05 (C), 111.76 (CH). HR-ESI-MS (*m*/*z*): calcd. for $C_{13}H_9Cl_2N_3$ 278.02463, found 278.02478 $[M+H]^+$.

5,6-dichloro-2-(4-(trifluoromethyl)phenyl)-1H-benzo[d]imidazole (**2j**)

Compound **2j** ($C_{14}H_7Cl_2F_3N_2$, MW 331.120) was obtained with a total yield of 68%; TLC (PS/EA 9/1): R_f 0.29. ^{1}H-NMR (DMSO-d_6): δ 8.40 (2H, d, J= 8 Hz, H-2',6'), 8.14 (2H, d, J= 8 Hz, H-3',5'), 7.98 (2H, s, H-4,7). ^{13}C-NMR (ATP, DMSO-d_6 +TFA-d): δ 150.92 (C), 135.52 (C), 131.47 (1C, q, 1J_{C-F}= 30 Hz, CF3), 130.11 (C), 128.11 (2CH), 126.87 (C), 126.82 (C), 126.08 (2C, $^3J_{C-F}$= 4 Hz, 2CH), 125.02 (C), 122.32 (C),116.19 (2CH). HR-ESI-MS (*m/z*): calcd. for $C_{14}H_7Cl_2F_3N_2$ 331.00111, found 331.00125 $[M+H]^+$.

5,6-dichloro-2-(p-tolyl)-1H-benzo[d]imidazole (**2k**)

Compound **2k** ($C_{14}H_{10}Cl_2N_2$, MW 277.149) was obtained with a total yield of 53%; TLC (PS/EA 7/3): R_f 0.45. ^{1}H-NMR (DMSO- d_6): δ 8.54 (2H, d, J= 8 Hz, H-2',6'), 8.19(2H, s, H-4,7), 7.27 (2H, d, J= 8 Hz, H-3',5'), 2.30 (3H, s, CH3). ^{13}C-NMR (ATP, DMSO- d_6 +TFA-d): δ 150.73 (C), 140.81 (C), 140.00 (C), 137.73 (C), 130.71 (C), 128.38 (2CH), 127.08 (C), 126.41 (2CH), 125.88 (C), 118.25 (2CH), 23.58 (CH_3). HR-ESI-MS (*m/z*): calcd. for $C_{14}H_{10}Cl_2N_2$ 277.02938, found 277.02945 $[M+H]^+$.

5,6-dichloro-2-(5-nitrothiophen-2-yl)-1H-benzo[d]imidazole (**2m**)

Compound **2m** ($C_{11}H_5Cl_2N_3O_2S$, MW 314.147) was obtained with a total yield of 58%; TLC (PS/EA 7/3): R_f 0.35. ^{1}H-NMR (DMSO-d_6): δ 8.18 (1H, d, J= 4.4 Hz, H-4'), 7.86 (2H, s, H-4,7), 7.82 (1H, d, J= 4.4 Hz, H-3'). ^{13}C-NMR (jmod, DMSO-d_6): δ 151.55 (C), 147.01 (C), 146.93 (C), 139.36 (2C), 130.89 (2CH), 126.79 (2CH), 125.77 (2C). HR-ESI-MS (*m/z*): calcd. for $C_{11}H_5Cl_2N_3O_2S$ 313.95523, found 313.95499 $[M+H]^+$.

5-chloro-2-(4-nitrophenyl)-1H-benzo[d]imidazole (**3b**)

Compound **3b** ($C_{13}H_8ClN_3O_2$, MW 273.674) was obtained with a total yield of 97%; %; TLC (PS/EA 7/3): R_f 0.44. ^{1}H-NMR (DMSO-d_6): δ 8.43 (4H, s, H-2',3',5',6'), 7.73 (1H, d, J=1.6 Hz, H-4), 7.69 (1H, d, J=8.4 Hz, H-7), 7.308 (1H, dd, ^1J=10.4 Hz, ^2J=2 Hz, H-6), 3.68 (1H, s, NH). ^{13}C-NMR (DMSO-d_6): δ 150.30 (C), 148.07 (C), 140.27 (C), 137.78 (C), 135.25 (C), 127.62 (2xCH), 127.36 (C), 124.31 (2xCH), 123.39 (CH), 116.70 (CH), 115.27 (CH). HR-ESI-MS (*m/z*): calcd. for $C_{13}H_8ClN_3O_2$ 274.037, found 274.037 $[M+H]^+$.

4-(5-chloro-1H-benzo[d]imidazol-2-yl)benzonitrile (**3c**)

Compound **3c** ($C_{14}H_8ClN_3$, MW 253.69) was obtained with a total yield of 99%; TLC (PS/EA 6/4): R_f 0.62. ^{1}H-NMR (DMSO-d_6): δ 8.38 (2H, d, J=8.4 Hz, H-2', 6'), 8.08 (2H, d, J=8.4 Hz, H-3',5'), 7.76 (1H, d, J_m=1.6 Hz, H-4), 7.71 (1H, d, J=8.4, H-7), 7.36 (1H, dd, J_o=8.4 Hz, J_m=1.6, 2.0 Hz, H-6). ^{13}C-NMR (DMSO-d_6): δ 150.69 (C), 139.33 (C), 136.93 (C), 133.55 (2xCH), 132.85 (C), 128.31 (C), 127.96 (2xCH), 124.34 (CH), 118.84 (C), 116.93 (CH), 115.45 (CH), 113.31 (C). ESI-MS *(m/z):* 254.1 $[M+H]^+$.

5-chloro-2-(4-isopropylphenyl)-1H-benzo[d]imidazole (**3d**)

Compound **3d** ($C_{16}H_{15}ClN_2$, MW 270.76) was obtained with a total yield of 75%; TLC (PS/EA 7/3): R_f 0.75. ^{1}H-NMR (DMSO-d_6): δ 8.12 (2H, d, J=8.4 Hz, H-2',6'), 7.66 (1H, d, J=1.6 Hz, H-4), 7.61 (1H, d, J=8.8 Hz, H-7), 7.40 (2H, d, J=8.4 Hz, H-3',5'), 7.19 (1H, dd, J_o=8.6 Hz, J_m=1.6 Hz, H-6), 2.95 (1H, q, CH aliphatic), 1.24 (6H, d, J=6.8 Hz, 2xCH_3). ^{13}C-NMR (DMSO-d_6): δ 152.90 (C), 150.61 (C), 127.46 (2xC), 126.86 (2xCH), 126.67 (2xCH), 126.06 (2xC), 121.97 (CH), 116.01 (CH), 114.85 (CH), 33.32 (CH), 15.18 (2xCH_3). HR-ESI-MS (*m/z*): calcd. for $C_{16}H_{15}ClN_2$ 271.099, found 271.100 $[M+H]^+$.

2-(4-(tert-butyl)phenyl)-5-chloro-1H-benzo[d]imidazole (**3e**)

Compound **3e** ($C_{17}H_{17}ClN_2$, MW 284.783) was obtained with a total yield of 83%; TLC (PS/EA 7/3): R_f 0.76. ^{1}H-NMR (acetone-d_6): δ 8.17 (2H, d, *J*=8.4 Hz, H-2',6'), 7.62-7.59 (4H, m, H-4,7,3',5'), 7.23 (1H, dd, ^1J=8.6 Hz, ^2J=1.6 Hz, 2 Hz, H-6), 1.38 (9H, s, 3xCH_3). ^{13}C-NMR (acetone-d_6): δ 154.37 (C), 153.81 (C), 141.61 (C), 139.11 (C), 128.10 (C), 128.07 (C), 127.40 (2xCH), 126.75 (2xCH), 126.35 (CH), 116.83 (CH), 115.80 (CH), 35.45 (C), 31.45 (3xCH_3). HR-ESI-MS (*m/z*): calcd. for $C_{17}H_{17}ClN_2$ 285.115, found 285.115 [M+H]$^+$.

4-(5-chloro-1H-benzo[d]imidazol-2-yl)aniline (**3i**)

Compound **3i** ($C_{13}H_{10}ClN_3$, MW 243.692) was obtained with a total yield of 86%; TLC: ($CHCl_3$/CH_3OH 7/3) R_f: 0.29. ^{1}H-NMR (DMSO-d_6): δ 7.83 (2H, d, *J*=8.4 Hz, H-2',6'), 7.54 (1H, m, H-4), 7.43 (1H, s, H-7), 7.13 (1H, d, *J*=8.4 Hz, H-6), 6.66 (2H, d, *J*=8.4 Hz), 5.66 (2H, s, NH_2). ^{13}C-NMR (DMSO-d_6+TFA-*d*): δ 153.63 (C), 150.93 (C), 132.17 (C), 130.24 (C), 129.65 (C), 129.60 (2xCH), 125.52 (CH), 114.42 (CH), 114.13 (2xCH), 112.76 (CH), 107.87 (C). HR-ESI-MS (*m/z*): calcd. for $C_{13}H_{10}ClN_3$ 244.063, found 244.063 [M+H]$^+$.

6-chloro-7-methyl-2-(4-nitrophenyl)-1H-benzo[d]imidazole (**4b**)

Compound **4b** ($C_{14}H_{10}ClN_3O_2$, MW 287.701) was obtained with a total yield of 78%; TLC (PS/EA 8/2): R_f 0.52. ^{1}H-NMR (DMSO-d_6): δ 8.37 (4H, s, H-2',3',5',6'), 7.52 (2H, d, J_o= 8.8 Hz, H-4), 7.36 (2H, d, J_o= 8.4 Hz, H-5), 2.59 (3H, s, CH_3). ^{13}C-NMR (jmod, DMSO-d_6): δ 149.10 (C), 148.42 (C), 138.49 (C), 135.02 (C), 133.61 (C), 128.44 (2CH), 127.92 (C), 124.83 (CH), 124.19 (2CH), 123.75 (C), 113.12 (CH), 14.53 (CH_3). HR-ESI-MS (*m/z*): calcd. for $C_{14}H_{10}ClN_3O_2$ 288.05343, found 288.05396 [M+H]$^+$.

2-(4-(tert-butyl)phenyl)-6-chloro-7-methyl-1H-benzo[d]imidazole (**4d**)

Compound **4d** ($C_{18}H_{19}ClN_2$, MW 298.8) was obtained with a total yield of 80%; TLC (PS/EA 8/2): R_f 0.64. ^{1}H-NMR (DMSO-d_6): δ 8.33 (2H, d, J=8.4 Hz, H-2',6'), 7.73 (2H, d, J=8.4 Hz, H-3,5), 7.63 (1H, d, J=8.8 Hz, H-5), 7.52 (1H, d, J= 8.8, H-4), 2.72 (GH, s, Ph-CH;), 1.36 (9H, s, 3CH_3). ^{13}C-NMR (jmod, DMSO-d_6): δ 156.0(C), 150.5 (2C),131.7(C), 129.3(C), 126.3 (2CH), 125.9 (1CH), 122.8 (C), 121.6 (C), 112.4 (CH), 33.5 (CH), 35.0 (C),30.8 (3CH_3), 14.7(CH_3). ESI-MS (*m/z*): 298.300 [M+H]$^+$.

6-chloro-2-(4-isopropylphenyl)-7-methyl-1H-benzo[d]imidazole (**4e**)

Compound **4e** ($C_{17}H_{17}ClN_2$, MW 284.78) was obtained with a total yield of 68%; TLC (PS/EA 8/2): R_f 0.46. ^{1}H-NMR (DMSO-d_6): δ (2H, d, J=6.0 Hz, H-2',6'), 7.63 (1H, d, J=6.6 Hz, H-5), 7.59 (2H, d, J=6.3 Hz, H-3',5'), 7.52 (1H, d, J=6.6, H-4), 3.50 (1H, sa, NH), 2.71(3H, s, Ph-CH;). ^{13}C-NMR (jmod, DMSO- d_6): δ 153.5 (2C), 150.8 (2C), 132.2(C),128.9(C), 128.1 (2CH), 127.4 (2CH), 125.6 (CH), 122.9(C), 112.5 (CH), 33.5 (CH), 25.45 (2CH_3),14.6(CH_3). ESI-MS (*m/z*): 284.1 [M+H]$^+$.

5,6-dimethyl-2-(4-nitrophenyl)-1H-benzo[d]imidazole (**5b**)

Compound 5b ($C_{15}H_{13}N_3O_2$, MW 267.283) was obtained with a total yield of 62%; %; TLC (PS/EA 7/3): R_f 0.43. ^{1}H-NMR (DMSO-d_6): δ 8.38 (4H, s, H-2',3',5',6'), 7.42 (2H, s, H-4,7), 2.34 (6H, s, 2CH_3). ^{13}C-NMR (DMSO-d_6): δ 147.92 (C), 147.52 (C), 137.75 (C), 135.79 (2C), 132.07 (2C), 128.85 (2CH), 127.69 (2CH), 124.19 (2CH), 19.96 (2CH_3). HR-ESI-MS (*m/z*): calcd. for $C_{15}H_{13}N_3O_2$ 268.10805, found 268.10797 [M+H]$^+$.

2-(4-isopropylphenyl)-5,6-dimethyl-1H-benzo[d]imidazole (**5d**)

Compound **5d** ($C_{18}H_{20}N_2$, MW 264.372) was obtained with a total yield of 31% %; TLC (PS/EA 6/4): R_f 0.76. 1H-NMR (DMSO-d_6): 8.14 (2H, d, J=8 Hz, H-2',6'), 7.52 (2H, d, J=8 Hz, H-3', 5'), 7.48 (2H, s, H-4,7), 3.00 (1H, ept, J=6.8 Hz, C-H),

2.37 (6H, s, 2 C-H_3), 1.27 (6H, d, J=6.8 Hz, 2 C-H_3). ^{13}C-NMR (DMSO-*d$_6$*): 152.22 (2C), 148.98 (2C), 133.74 (C), 133.06 (C), 127.23 (2CH), 127.13 (2CH), 124.12 (C), 114.14 (2CH), 33.42 (CH), 23.64 (CH_3), 23.52 (CH_3), 19.94 (2CH_3). ESI-MS (*m*/*z*): 264.8 $[M+H]^+$.

2-(4-(tert-butyl)phenyl)-5,6-dimethyl-1H-benzo[d]imidazole (**5e**)

Compound **5e** ($C_{19}H_{22}N_2$, MW 278.399) was obtained with a total yield of 35%; TLC (PS/EA 6/4): R_f 0.8. ^{1}H-NMR (DMSO-*d$_6$*): 8.25 (2H, d, J=8 Hz, H-2',6'), 7.74 (2H, d, J=8 Hz, H-3', 5'), 7.59 (2H, s, H-4,7), 2.41 (6H, s, 2 CH_3), 1.27 (9H, s, 2 CH_3). 13C-NMR (DMSO-*d$_6$*): 156.57 (2C), 148.24 (2C), 135.54 (2C), 131.17 (C), 128.04 (2CH), 126.93 (2CH), 121.37 (C), 114.01 (2CH), 35.47 (CH), 31.20 (3CH_3), 20.39 (2CH_3). ESI-MS (*m*/*z*): 279.1 $[M+H]^+$.

4-(5,6-dimethyl-1H-benzo[d]imidazol-2-yl)aniline (**5i**)

Compound **5i** ($C_{15}H_{15}N_3$, MW 237.299) was obtained with a total yield of 88%; TLC: ($CHCl_3$/CH_3OH 95/5) R_f: 0.17. ^{1}H-NMR (DMSO-*d$_6$*): *δ* 7.80 (2H, d, J= 8.4 Hz, H-2',6'), 7.31 (1H, s, H-7), 7.18 (1H, s, H-4), 6.65 (2H, d, J= 8.8 Hz, H-3',5'), 5.55 (2H, s, NH2), 2.29 (6H, d, J= 7.2 Hz). ^{13}C-NMR (DMSO-*d$_6$*): *δ* 151.62 (C), 150.21 (C), 137.32 (2C), 130.01 (2C), 127.52 (2CH), 117.13 (2C), 114.37 (C), 113.78 (2CH), 19.84 (2CH_3). HR-ESI-MS (*m*/*z*): calcd. for $C_{15}H_{15}N_3$ 238.13387, found 238.13365 $[M+H]^+$.

5-methyl-2-(4-nitrophenyl)-1H-benzo[d]imidazole (**6b**)

Compound **6b** ($C_{14}H_{11}N_3O_2$, MW 253.256) was obtained with a total yield of 42%; TLC: ($CHCl_3$/CH_3OH 95/5) R_f: 0.74. ^{1}H-NMR (DMSO-*d$_6$*): *δ* 8.57 (2H, d, *J*=9.2 Hz, H-3',5'), 8.52 (2H, d, *J*=8.8 Hz, H-2',6'), 7.73 (1H, d, *J*=8.4 Hz, H-7), 7.63 (1H, s, H-4), 7.37 (1H, d, *J*=8.4 Hz), 2.50 (3H, s, CH_3). ^{13}C-NMR (DMSO-*d$_6$*): *δ* 149.13 (C), 146.57 (C), 135.75 (C), 133.82 (C), 131.98 (C), 130.62 (C), 128.91 (2xCH), 127.32 (CH), 124.48 (2xCH), 114.28 (CH), 113.76 (CH), 21.23 (CH_3). HR-ESI-MS (*m*/*z*): calcd. For $C_{14}H_{11}N_3O_2$ 254.092, found 254.092 $[M+H]^+$.

4-(5-methyl-1H-benzo[d]imidazol-2-yl)benzonitrile (**6c**)

Compound **6c** ($C_{15}H_{11}N_3$, MW 233.27) was obtained with a total yield of 48%; TLC (PS/EA 6/4): R_f 0.52. ^{1}H-NMR (DMSO-*d$_6$*): δ 8.33 (2H, d, *J*=8.4 Hz, H-2',6'), 8.03 (2H, d, *J*=8.4 Hz, H-3',5'), 7.56 (1H, d, *J*=8.4 Hz, H-7), 7.45 (1H, s, H-4), 7.12 (1H, d, *J*=8.4 Hz, H-6), 2.45 (3H, s, CH_3). ^{13}C-NMR (DMSO-*d$_6$*): δ 149.16 (C), 138.64 (C), 137.62 (C), 133.97 (C), 133.43 (2 CH), 133.22 (C), 127.49 (2xCH), 125.26 (CH), 119.01 (C), 115.87 (CH), 114.90 (CH), 112.50 (C), 20.71 (CH_3). HR-ESI-MS (*m*/*z*): calcd. for $C_{15}H_{11}N_3$ 233.27, found 234.102 $[M+H]^+$.

2-(4-isopropylphenyl)-5-methyl-1H-benzo[d]imidazole (**6d**)

Compound **6d** ($C_{17}H_{18}N_2$, MW 250.35) was obtained with a total yield of 69%; TLC (PS/EA 6/4): R_f 0.71. ^{1}H-NMR (DMSO-*d$_6$*): δ 8.29 (2H, d, *J*=8.4 Hz, H-2', 6'), 7.68 (1H, d, *J*=8.4 Hz, H-6), 7.57 (3H, m, H-4, 3', 5'), 7.32 (1H, d, *J*=8.4, H-7), 3.018 (1H, ept, *J*=6.8 Hz CH_3), 2.49 (3H, s, CH_3), 1.26 (6H, d, *J*=6.8 Hz, 2 CH_3). ^{13}C-NMR (DMSO-*d$_6$*): δ 153.74 (C), 148.63 (C), 135.18 (C), 132.93 (C), 130.92 (C), 127.85 (2xCH), 127.50 (2xCH), 126.75 (CH), 121.66 (C), 113.64 (CH), 113.37 (CH), 33.50 (CH), 23.40 (2xCH_3), 21.17 (CH_3). ESI-MS (*m*/*z*): 253.9 $[M+H]^+$.

2-(4-(tert-butyl)phenyl)-5-methyl-1H-benzo[d]imidazole (**6e**)

Compound **6e** ($C_{18}H_{20}N_2$, MW 264.37) was obtained with a total yield of 58%; TLC (PS/EA 6/4): R_f 0.79. ^{1}H-NMR (DMSO-*d$_6$*): δ 8.32 (2H, d, *J*=8.4 Hz, H-2', 6'), 7.745-7.645 (3H, m, H-3',5', 7), 7.61 (1H, s, H-4), 7.36 (1H, d, *J*=8.4, H-6), 2.50 (3H, s, CH_3), 1.35 (3H, s, 3xCH_3). ^{13}C-NMR (DMSO-*d$_6$*): δ 156.28 (C), 148.33 (C), 132.34 (C), 129.04 (C), 127.73 (2xCH),

127.10 (CH), 126.46 (2xCH), 120.72 (C), 113.53 (CH), 34.99 (C), 30.79 (3xCH_3), 21.16 (CH_3). HR-ESI-MS (*m/z*): calcd. for $C_{18}H_{20}N_2$ 264.37, found 265.170 $[M+H]^+$.

4-(5-methyl-1H-benzo[d]imidazol-2-yl)aniline (**6i**)

Compound **6i** ($C_{14}H_{13}N_3$, MW 223.273) was obtained with a total yield of 31%; TLC: ($CHCl_3$/CH_3OH 95/5) R_f: 0.26. ^{1}H-NMR (acetone-d_6): δ 7.94 (2H, d, *J*=8.8 Hz, H-2',6'), 7.39 (1H, d, *J*=8 Hz, H-7), 7.31 (1H, s, H-4), 6.97 (1H, d, *J*=8 Hz, H-6), 6.78 (2H, d, *J*=8.4 Hz, H-3',5'), 5.09 (2H, s, NH_2), 2.42 (3H, s, CH_3). ^{13}C-NMR (DMSO-d_6+ TFA-*d*): δ 153.69 (C), 149.49 (C), 135.10 (C), 131.54 (C), 129.33 (2xCH), 129.11 (C), 126.45 (CH), 114.07 (2xCH), 113.42 (2xCH), 108.04 (C), 20.97 (CH_3). HR-ESI-MS (*m/z*): calcd. for $C_{14}H_{13}N_3$ 224.118, found 224.117 $[M+H]^+$.

5,6-difluoro-2-(4-nitrophenyl)-1H-benzo[d]imidazole (**7b**)

Compound **7b** ($C_{13}H_7F_2N_3O_2$, MW 275.21) was obtained with total yield of 52%; TLC (PS/EA 7/3): R_f 0.40. ^{1}H-NMR (DMSO- d_6): δ 8.39 (4H, m, H-2',3',5',6'), 7.715 (2H, t, H-4,7). ^{13}C-NMR (jmod, DMSO- d_6): δ 149.70 (C), 148.88 (C), 148.39 (2C, dd, $^1J_{C-F}$= 245 Hz, $^2J_{C-F}$= 19 Hz, 2C-F), 131.84 (C, m), 131.66 (C), 131.25 (C, m), 128.35 (2CH), 124.28 (2CH), 102.90 (2C, dd, $^2J_{C-F}$= 15 Hz, $^3J_{C-F}$= 8 Hz, CH-4,7). HR-ESI-MS (*m/z*): calcd. for $C_{13}H_7F_2N_3O_2$ 276.05791, found 276.05856 $[M+H]^+$.

5,6-difluoro-2-(4-isopropylphenyl)-1H-benzo[d]imidazole (**7d**)

Compound **7d** ($C_{16}H_{14}F_2N_2$, MW 272.293) was obtained with a total yield of 67%; TLC (PS/EA 7/3): R_f 0.75. ^{1}H-NMR (DMSO- d_6): δ 8.17 (d, 2H, J= 8.4 Hz, H-2',6'), 7.82 (t, 2H, J= 8.8 Hz, H-4,7), 7.54 (d, 2H, J= 8 Hz, H-3',5'), 3.01 (m, 1H, J= 6.8 Hz, CH), 1.26 (d, 6H, J= 6.8 Hz, 2CH_3). ^{13}C-NMR (jmod, DMSO- d_6): δ 153.24 (C), 151.67 (C), 147.94 (2C, dd, $^1J_{C-F}$= 242 Hz, $^2J_{C-F}$= 16 Hz, 2C-F), 130.26 (2C), 127.57 (2CH), 127.41 (2CH), 122.94 (C), 102.52 (2CH, m, CH-4,7), 33.48 (CH), 23.45 (2CH_3). HR-ESI-MS (*m/z*): calcd. for $C_{16}H_{14}F_2N_2$ 273.11978, found 273.11948 $[M+H]^+$.

2-(4-(tert-butyl)phenyl)-5,6-difluoro-1H-benzo[d]imidazole (**7e**)

Compound 7e ($C_{17}H_{16}F_2N_2$, MW 286.319) was obtained with a total yield of 78%; TLC (PS/EA 7/3): R_f 0.78. ^{1}H-NMR (DMSO- d_6): δ 8.14 (d, 2H, J= 8.4 Hz, H-2',6'), 7.78 (t, 2H, J= 8.8 Hz, H-4,7), 7.66 (d, 2H, J= 8.4 Hz, H-3',5'), 1.35 (s, 9H, 3CH_3). ^{13}C-NMR (jmod, DMSO- d_6): δ 154.65 (C-2), 152.25 (C-4'), 147.56 (2C, dd, $^1J_{C-F}$= 241 Hz, $^2J_{C-F}$= 16 Hz, 2CF), 131.75 (C-3a,7a), 126.86 (C-2',6'), 126.15 (C-3',5'), 123.97 (C-1'), 102.66 (2CH, dd, $^2J_{C-F}$= 15 Hz, CH-4,7), 34.80 (C), 30.81 (3CH_3). HR-ESI-MS (*m/z*): calcd. for $C_{17}H_{16}F_2N_2$ 287.13543, found 287.13599 [M+H]+.

4-(5,6-difluoro-1H-benzo[d]imidazol-2-yl)aniline (**7i**)

Compound **7i** ($C_{13}H_7F_2N_3O_2$, MW 245.227) was obtained with a total yield of 87%; TLC: ($CHCl_3$/CH_3OH 95/5) R_f: 0.40.^{1}H-NMR (DMSO- d_6): δ 7.81 (2H, d, J= 8.4 Hz, H-2',6'), 7.54 (1H, s, H-4), 7.448 (1H, s, H-7), 6.67 (2H, d, J= 8.8 Hz, H-3',5'), 5.64 (2H, s, NH2). ^{13}C-NMR (jmod, DMSO- d_6): δ 154.62 (C), 150.85 (2C), 146.21 (2C, dd, $^1J_{C-F}$= 236 Hz, $^2J_{C-F}$= 16 Hz, C-F), 127.75 (2CH), 116.53 (2C), 113.51 (2CH), 102.45-101.30 (2C, m, CH-F). HR-ESI-MS (*m/z*): calcd. for $C_{13}H_7F_2N_3O_2$ 246.08373, found 246.08331 $[M+H]^+$.

5-fluoro-2-(4-nitrophenyl)-1H-benzo[d]imidazole (**8b**)

Compound **8b** ($C_{13}H_8FN_3O_2$, MW 257.22) was obtained with a total yield of 87%; TLC (PS/EA 7/3): R_f 0.45. ^{1}H-NMR (DMSO-d_6): δ 8.45 (4H, d, *J*=1.6 Hz, H-2',3',5',6'), 7.74 (1H, m, H-7'), 7.45 (1H, m, H-4'), 7.23 (1H, m, H-6'). ^{13}C-NMR (DMSO-d_6): δ 160.54 (C), 158.18 (C), 149.72 (C), 148.35 (C), 137.91 (C), 133.97 (C), 127.95 (2xCH), 124.36 (2xCH), 116.44 (CH), 112.26 (CH), 101.29 (CH). HR-ESI-MS (*m/z*): calcd. for $C_{13}H_8FN_3O_2$ 258.067, found 258.067 $[M+H]^+$.

5-fluoro-2-(4-isopropylphenyl)-1H-benzo[d]imidazole (**8d**)

Compound **8d** ($C_{16}H_{15}FN_2$, MW 254.302) was obtained with a total yield of 76%; TLC (PS/EA 7/3): R_f0.56. ^{1}H-NMR (acetone-d_6): δ 8.14 (2H, d, J=8 Hz, H-2',6'), 7.58 (1H, m, H-4), 7.44 (2H, d, J=8 Hz, H-3',5'), 7.32 (1H, d, J_o=8 Hz), 7.02 (1H, ddd, J_{para}=1.8 Hz, J_{ortho}=8Hz, $^3J_{H-F}$=11.4 Hz, H-6), 3.01 (1H, q, J=7 Hz, CH-aliphatic, 1.30 (6H, d, J=7.2 Hz, 2xCH$_3$). ^{13}C-NMR (DMSO-d_6): δ 159.78 (C), 157.44 (C), 152.72 (C), 150.80 (C), 137.47 (1C, d, $^1J_{C-F}$=402 Hz, C-F), 127.12 (C), 126.96 (2xCH), 126.50 (2xCH), 115.45 (CH-7), 110.17 (1C, d, $^2J_{C-F}$=26 Hz, CH-6), 100.99 (1C, d, $^2J_{C-F}$= 25 Hz, CH-4), 33.29 (CH), 23.56 (2xCH$_3$). HR-ESI-MS (*m/z*): calcd. for $C_{16}H_{15}FN_2$ 255.129, found 255.128 [M+H]$^+$.

2-(4-(tert-butyl)phenyl)-5-fluoro-1H-benzo[d]imidazole (**8e**)

Compound **8e** ($C_{17}H_{17}FN_2$, MW 268.329) was obtained with a total yield of 84%; TLC (PS/EA 7/3): R_f0.72. ^{1}H-NMR (DMSO-d_6): δ 8.09 (2H, d, J=8 Hz, H-2',6'), 7.57 (3H, d, J=8.4 Hz, H-4,3',5'), 7.38 (1H, d, J=8.4 Hz, H-7), 7.05 (1H, m, H-6), 1.33 (9H, s, 3xCH$_3$). ^{13}C-NMR (DMSO-d_6): δ 159.83 (C), 157.50 (C), 153.22 (C), 152.60 (C), 137.22 (1C, d, $^1J_{C-F}$=401 Hz, C-F), 126.44 (C), 126.28 (2xCH), 125.87 (2xCH), 115.50 (1C, d, $^3J_{C,F}$=10 Hz, CH-7), 110.38 (1C, $^2J_{C,F}$=25 Hz, CH-6), 100.95 (1C, $^2J_{C,F}$=26 Hz, CH-4), 34.55 (C), 30.84 (3xCH$_3$). HR-ESI-MS (*m/z*): calcd. for $C_{17}H_{17}FN_2$ 269.144, found 269.144 [M+H]$^+$.

4-(5-fluoro-1H-benzo[d]imidazol-2-yl)aniline (**8i**)

Compound **8i** ($C_{13}H_{10}FN_3$, MW 227.237) was obtained with a total yield of 63%; TLC (PS/EA 7/3): R_f0.12. ^{1}H-NMR (DMSO-d_6): δ 8.05 (2H, d, J=8.8 Hz, H-2',6'), 7.72 (1H, m, H-7), 7.54 (1H, m, H-4), 7.40 (1H, m, H-6), 6.78 (2H, d, J=8.8 Hz, H-3',5').^{13}C-NMR (DMSO-d_6): δ 160.81 (C), 158.42 (C), 153.90 (C), 151.15 (C), 132.42 (C), 129.67 (2xCH), 128.48 (C), 114.43 (CH), 113.66 (2xCH), 113.02 (CH), 99.95 (CH). HR-ESI-MS (*m/z*): calcd. for $C_{13}H_{10}FN_3$ 228.093, found 228.093 [M+H]$^+$.

4-fluoro-2-(4-nitrophenyl)-1H-benzo[d]imidazole (**9b**)

Compound **9b** ($C_{13}H_8FN_3O_2$,MW 257.220) was obtained with a total yield of 65%; TLC (PS/EA 7/3): R_f 0.81. ^{1}H-NMR (DMSO- d_6): δ 8.45 (4H, dd, J= 9.2 Hz, H-2',3',5',6'), 7.47 (1H, d, J_{ortho}= 8 Hz, H-7), 7.28 (1H, td, J_{ortho}= 8.1 Hz, $J_{meta\ H-F}$= 5.1 Hz, H-6), 7.09 (1H, m, H-5). ^{13}C-NMR (jmod, DMSO- d_6): δ 153.46 (C), 150.98 (C), 149.64 (C), 148.08 (C), 139.71 (1C, m, C-F), 135.17 (C), 127.71 (2CH), 124.29 (2CH), 123.99 (1C, d, $^3J_{C-F}$= 7 Hz, CH-6), 109.81 (CH-7), 107.83 (1C, d, $^2J_{C-F}$= 16 Hz, CH-5). HR-ESI-MS (*m/z*): calcd. for $C_{13}H_8FN_3O_2$ 258.06733, found 258.06766 [M+H]$^+$.

4-(3H-imidazo[4,5-b]pyridin-2-yl)benzonitrile (**10c**)

Compound **10c** ($C_{13}H_8N_4$, MW 220.23) was obtained with a total yield of 4%; TLC: ($CHCl_3$/CH_3OH 95/5) R_f 0.43. ^{1}H NMR (DMSO-d_6 + TFA-d): δ 8.75-8.70 (2H, m, H-5, 7), 8.45 (2H, d, J=8.4 Hz, H-2', 6'), 8.14 (2H, d, J=7.2 Hz, H-3', 5'), 7.77 (1H, m, H-6). ^{13}C NMR (DMSO-d_6 + TFA-d): δ 155.99 (C), 148.24 (C), 137.84 (CH), 133.66 (2CH), 131.91 (C), 131. 65 (C), 129.27 (CH), 128.86 (2CH), 119.79 (CH), 118.43 (C), 115.01 (C). ESI-MS (*m/z*): 221.2 [M+H]$^+$.

2-(4-(trifluoromethyl)phenyl)-3H-imidazo[4,5-b]pyridine (**10j**)

Compound **10j** ($C_{13}H_8F_3N_3$, MW 263.22) was obtained with a total yield of 12%; TLC: ($CHCl_3$/CH_3OH 95/5) R_f 0.43. ^{1}H NMR (DMSO-d_6 + TFA-d): δ 8.74-8.65 (2H, m, H-5, 7), 8.49 (2H, d, J=8.4 Hz, H-3', 5'), 8.03 (2H, d, J=8 Hz, H-2',6'), 7.78-7.74 (1H, m, H-6). ^{13}C NMR (DMSO-d_6 + TFA-d): δ 156.36 (C), 148.15 (C), 137.52 (C-H), 132.71 ($^2J_{C-F}$=32 Hz, CF$_3$), 131.72 (2C), 131.17 (C), 129.10 (CH), 129.04 (2CH), 124.04 ($^1J_{C-F}$=271 Hz, CF$_3$), 126.65 (CH), 126.62 (C-H). ESI-MS (*m/z*): 263.6 [M+H]$^+$.

2-(3-fluorophenyl)-3H-imidazo[4,5-b]pyridine (**10h**)

Compound **10h** ($C_{12}H_8N_3F$, MW 213.215) was obtained with a total yield of 59%; TLC: ($CHCl_3/CH_3OH$ 95/5) R_f 0.55. ^{1}H-NMR (400 MHz, DMSO-*d6*) δ 8.37 (s, 1H, H-2'), 8.10-8.01 (m, 3H, H-5, 7, 6'), 7.64 (m, 1H, H-4'), 7.39 (td, J_{H-H}= 8.7, $J=_{H-F\ meta}$ 2.6 Hz, 1H, H-5'), 7.28 (dd, *J* = 8.0, 4.8 Hz, 1H, H-6). ^{13}C-NMR (101 MHz, DMSO-*d6*) δ 162.87 (d, $^1J_{C-F}$ = 245.43 Hz, C) ,144.72 (CH), 132.44 (C), 132.36 (C), 131.75 (CH), 131.67 (CH), 123.29 (CH), 118.82 (CH), 117.76 (d, $^2J_{C-F}$ = 21.21 Hz, CH), 113.75 (d, $^2J_{C-F}$= 24.24 Hz, CH). ESI-MS (*m/z*): 214.3 [M+H]$^+$.

2-(4-nitrophenyl)-3H-imidazo[4,5-c]pyridine (**11b**)

Compound **11b** ($C_{12}H_8N_4O_2$, MW 240.221) was obtained with a total yield of 46%; TLC: ($CHCl_3/CH_3OH$ 95/5) R_f: 0.22. ^{1}H NMR (DMSO-d_6 + TFA-*d*): δ 9.58 (1H, s, H-4), 8.66 (1H, d, J=6.4 Hz, H-6), 8.52 (2H, d, J=8.8 Hz, H-3', 5'), 8.40 (2H, d, J=9.2 Hz, H-2', 6'), 8.24 (1H, d, J=6.4 Hz, H-7). ^{13}C NMR (DMSO-d_6 + TFA-*d*): δ 156.64 (C), 149.28 (C), 146.41 (C), 138.99 (C), 133.93 (CH), 133.80 (CH), 133.38 (C), 129.46 (2CH), 124.22 (2CH), 111.19 (CH). ESI-MS (*m/z*): 241.0 [M+H]$^+$.

4-(3H-imidazo[4,5-c]piridin-2yl)benzonitrile (**11c**)

Compound **11c** ($C_{13}H_8N_4$, MW 220.23) was obtained with a total yield of 12%; TLC: ($CHCl_3/CH_3OH$ 95/5) R_f 0.23. ^{1}H NMR (DMSO-d_6 + TFA-*d*): δ 9.55 (1H, s, H-4), 8.65 (1H, d, J=6 Hz, H-6), 8.45 (2H, d, J=8 Hz, H-2', 6'), 8.22 (1H, d, J=6 Hz, H-7), 8.12 (2H, d, J=6-8 Hz, H-3', 5'). ^{13}C NMR (DMSO-d_6 + TFA-*d*): δ 157.05 (C), 146.44 (C), 138.95 (C), 135.99 (CH), 133.69 (2CH), 133.09 (2CH), 131.73 (C), 128.29 (2CH), 117.99 (C), 114.23 (C), 111.07 (CH). ESI-MS (*m/z*): 220.7 [M+H]$^+$.

2-(3-fluorophenyl)-3H-imidazo[4,5-c]pyridine (**11h**)

Compound **11h** ($C_{12}H_8N_3F$, MW 213.215) was obtained with a total yield of 63%; TLC: ($CHCl_3/CH_3OH$ 95/5) R_f 0.26. ^{1}H-NMR (400 MHz, DMSO-*d6*) δ 8.98 (s, 1H, H-2'), 8.33 (d, J = 5.6 Hz, 1H, H-6), 8.10 (d, J = 7.8 Hz, 1H, H-7), 8.04 (dt, $J_{H-F\ ortho}$ = 10.1, J_{H-H}= 2.1 Hz, 1H, H-2'), 7.69 – 7.59 (m, 2H, H-4',6'), 7.40 (td, J_{H-H}= 8.5, $J=_{H-F\ meta}$ 2.7 Hz, 1H, H-5'). ^{13}C-NMR – Katholieke Universiteit Leuven. (2021). Pyrrolopyridine and imidazopyridine antiviral compounds (WO n. WO2021/170600 A1). World Intellectual Property Organization. (Patent). ESI-MS (*m/z*): 214.1 [M+H]$^+$.

2-(2-fluorophenyl)-3H-imidazo[4,5-c]pyridine (**11l**)

Compound **11l** ($C_{12}H_8N_3F$, MW 213.215) was obtained with a total yield of 62%; TLC: ($CHCl_3/CH_3OH$ 95/5) R_f 0.3. ^{1}H-NMR (400 MHz, DMSO-*d6* + TFA-*d*) δ 9.51 (s, 1H, H-4), 8.67 – 8.59 (m, 1H, H-6), 8.31 (td, J = 7.7, 1.8 Hz, 1H, H-4'), 8.21 – 8.14 (m, 1H, H-3'), 7.77-7.70 (m, 1H, H-5'), 7.59 – 7.45 (m, 2H, H-7, H-6'). ^{13}C-NMR (101 MHz, DMSO-*d6* + TFA-*d*) δ160,49 (d, $^1J_{C-F}$= 254.52 Hz, C), 154.63 (C), 146.89 (C), 138.84 (C), 134.81 (d, $^3J_{C-F}$ = 9.09 Hz, CH), 134.03(CH), 133.78 (CH), 131.33 (d, $^4J_{C-F}$ = 2.02 Hz, CH), 125.93 (CH, d, J_{C-F} = 4.04), 117.3 (d, $^2J_{C-F}$ = 22.22 Hz, CH), 116.31 (d, $^3J_{C-F}$= 11.11 Hz, C), 111.71 (CH). ESI-MS (*m/z*): 214.3 [M+H]$^+$.
